# Global response of diacylglycerol kinase towards substrate binding observed by 2D and 3D MAS NMR

**DOI:** 10.1038/s41598-019-40264-8

**Published:** 2019-03-08

**Authors:** Kristin Möbius, Sina Kazemi, Peter Güntert, Andreas Jakob, Alexander Heckel, Johanna Becker-Baldus, Clemens Glaubitz

**Affiliations:** 1 Institute of Biophysical Chemistry & Centre for Biomolecular Magnetic Resonance, Goethe-University Frankfurt, Frankfurt, Germany; 2 Laboratory of Physical Chemistry, ETH Zürich, Zürich, Switzerland; 3 Graduate School of Science, Tokyo Metropolitan University, Tokyo, Japan; 4 Institute of Chemical Biology and Organic Chemistry, Goethe-University Frankfurt, Frankfurt, Germany

## Abstract

*Escherichia coli* diacylglycerol kinase (DGK) is an integral membrane protein, which catalyses the ATP-dependent phosphorylation of diacylglycerol (DAG) to phosphatic acid (PA). It is a unique trimeric enzyme, which does not share sequence homology with typical kinases. It exhibits a notable complexity in structure and function despite of its small size. Here, chemical shift assignment of wild-type DGK within lipid bilayers was carried out based on 3D MAS NMR, utilizing manual and automatic analysis protocols. Upon nucleotide binding, extensive chemical shift perturbations could be observed. These data provide evidence for a symmetric DGK trimer with all of its three active sites concurrently occupied. Additionally, we could detect that the nucleotide substrate induces a substantial conformational change, most likely directing DGK into its catalytic active form. Furthermore, functionally relevant interprotomer interactions are identified by DNP-enhanced MAS NMR in combination with site-directed mutagenesis and functional assays.

## Introduction

**Figure 1:**
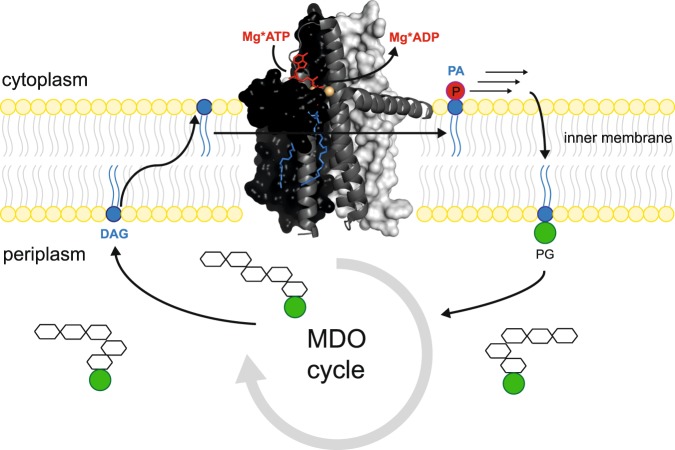
Physiological role of *E.coli* diacylglycerol kinase (DGK) in recycling during the biosynthesis of membrane-derived oligosaccharides (MDOs)^1,2^. MDOs are largely generated in response to environmental stress, such as low osmolarity^4,5^. DGK is located within the inner membrane, where it catalyzes the ATP-dependent phosphorylation of potentially membrane-disruptive diacylglycerol (DAG) to non-toxic phosphatic acid (PA). It provides the basis for restoring phosphatidylglycerol (PG), which is consumed in the MDO cycle. The cartoon is based on the X-ray structure using the PDB ID 4UXX^11^.

*Escherichia coli* diacylglycerol kinase (DGK) is a homotrimeric enzyme encoded by the dgkA gene. It is located within the inner membrane, where it catalyses the ATP-dependent phosphorylation of diacylglycerol (DAG) to phosphatic acid (PA) at the membrane interface. It plays an important role in recycling DAG during the biosynthesis of membrane-derived oligosaccharides (MDOs) (Fig. 1)^1,2^ and lipopolysaccharides (LPSs)^3^. MDOs are largely generated in response to environmental stress, such as low osmolarity^4,5^, whereas LPSs are the main constituent of the bacterial outer membrane^3^. *E.coli* DGK is a unique enzyme. It has no significant homology with other proteins except for undecaprenol kinase (UDPK) present in Gram-positive bacteria. It does not feature a P-loop or any other structural or functional motif that is widespread among water-soluble kinases. It is different from the eukaryotic diacylglycerol kinases and their prokaryotic homologs, encoded by the dgkB gene^6–9^. With 43 kDa (121 residues per monomer), DGK is the smallest known kinase^9^. It features a notable complexity in structure and function^9–12^ as well as a remarkable stability^13,14^. DGK forms a homotrimer, in which each monomer contains three transmembrane helices (H1-3) and one N-terminal amphiphilic surface helix (SH)^10,12,15^. The trimer contains three active sites near the membrane/cytoplasm interface. Each active site is formed by the transmembrane region of one monomer and the surface helix of an adjacent monomer leading to an unusual catalytic site architecture of the composite shared site model^10,11,16^. This high complexity in combination with a convenient experimental handling has put DGK into focus as a model system for investigations of membrane protein structure, function and folding^6,9,15–20^.

So far, two 3D structures have been published for DGK: one obtained by solution NMR in dodecylphosphocholine (DPC) micelles^12^ and one by X-ray crystallography in lipidic cubic phases (LCP) composed of monoolein, which also acts as a lipid substrate^10^. In addition, also a crystal structure of the thermostable mutant, Δ4-DGK (I53C, I70L, M96L, V107D), with bound lipid substrate and a co-crystallized ATP analogue^11^ was determined. Caffrey and co-workers could identify several residues interacting specifically with the nucleotide or lipid substrate, proposing a catalytic mechanism^11^. However, a number of mechanistic questions remain unsolved. It is unknown yet whether the three active sites of DGK are in the same or different states during catalysis and whether DGK undergoes a substantial conformational change prior to the actual phosphoryl transfer. Taking into account that the DGK trimer exhibits a remarkable stability and that each active site is built by components of two protomers based on the composite shared site model, the question arises whether specific long-range intra- and interprotomer interactions exist.

Our previous studies on DGK focused on its catalytic activity at the membrane interface by real-time ^31^P-MAS NMR^21^. Here, we address the questions defined above by multidimensional ^13^C-^15^N-MAS NMR as well as by dynamic nuclear polarisation. The key advantage of solid-state NMR and in particular of MAS is the possibility of performing experiments directly within the lipid bilayer^22^ or even within the cellular context^23^, which brings it closer to physiological conditions compared to other membrane-mimicking environments. The membrane environment is of key importance as it is a strong structural factor. It is also directly linked to the catalytic activity of DGK and many other membrane proteins^24–27^. Using solid-state NMR, full resonance assignments of membrane proteins in lipid bilayers^28–30^, but also 3D structure determination has been demonstrated^31^. Furthermore, the dramatic signal enhancement provided by DNP^32,33^ enabled new, mainly hypothesis-driven applications, which would not be possible by conventional solid-state NMR^34–37^.

Here, we used multidimensional MAS NMR for the ^13^C- and ^15^N-sequential assignment of uniformly labelled DGK prepared in its apo- and nucleotide-bound states. High-quality spectra could be obtained, enabling 84% resonance assignment, which could be verified to a high extent by the automatic assignment algorithm ssFLYA^38^. Chemical shift perturbations and altered peak intensities were analysed in 3D heteronuclear correlation spectra upon binding of the ATP analogue adenylylmethylenediphosphonate (AMP-PCP). Our data demonstrate that the global response of membrane proteins in a lipid environment towards substrate binding could be probed. We are able to highlight changes in structure and dynamics within the catalytic hotspots of DGK, providing a deeper understanding of its catalytic mechanism. Our data give evidence that all three active sites of DGK can be occupied by the nucleotide substrate at the same time. Additionally, a substantial conformational change prior to the phosphoryl transfer induced by the nucleotide substrate could be detected. In addition, the effect of the lipid substrate *sn*-1,2-dioctanoylglycerol (DOG) has been probed. Moreover, functionally relevant intra- and interprotomer contacts could be visualized in conventional high-field MAS NMR spectra and especially through experiments utilizing sensitivity enhancement by dynamic nuclear polarisation (DNP).

## Results

All MAS NMR experiments described here were carried out on wild-type DGK embedded within lipid bilayers as explained in Material and Methods. Sample purity was tested by SDS-PAGE (Suppl. Fig. 1a), trimer formation was verified by BN-PAGE (Suppl. Fig. 1b), and homogenous reconstitution was shown by density gradient centrifugation (Suppl. Fig. 1c). As liposome composition 90 mol% DMPC/10 mol% DMPA was used, which has been already successfully applied for several membrane proteins^31,36,39–41^. High quality MAS NMR spectra of DGK with good resolution could be obtained (Suppl. Fig. 2a,b) and the effect of different lipid compositions on the spectral fingerprint and activity of DGK has been probed (Suppl. Fig. 3). In order to obtain well-resolved spectra, all NMR experiments were conducted at 4 °C, which minimizes sample dehydration and provides better CP efficiency of the protein sample, since the surrounding lipids are in the gel phase^42^. Suppl. Fig. 2c illustrates the superposition of the PDSD spectrum with the resonance assignment of the thermostable DGK mutant, published by Yang and co-workers^29^. Though this assignment was obtained using MAS NMR as well, significant deviations made a *de-novo* assignment of wild-type DGK necessary. Deviations are most likely caused by inserted mutations and/or different experimental conditions. Our experiments were carried out on DGK reconstituted into DMPC/DMPA under a pH of 7.2 and full hydration. The thermostable mutant was reconstituted into *E.coli* total lipids and measured under a pH of 6.6 and 23 wt% H_2_O^29^.

### Resonance assignment and secondary structure analysis

**Figure 2:**
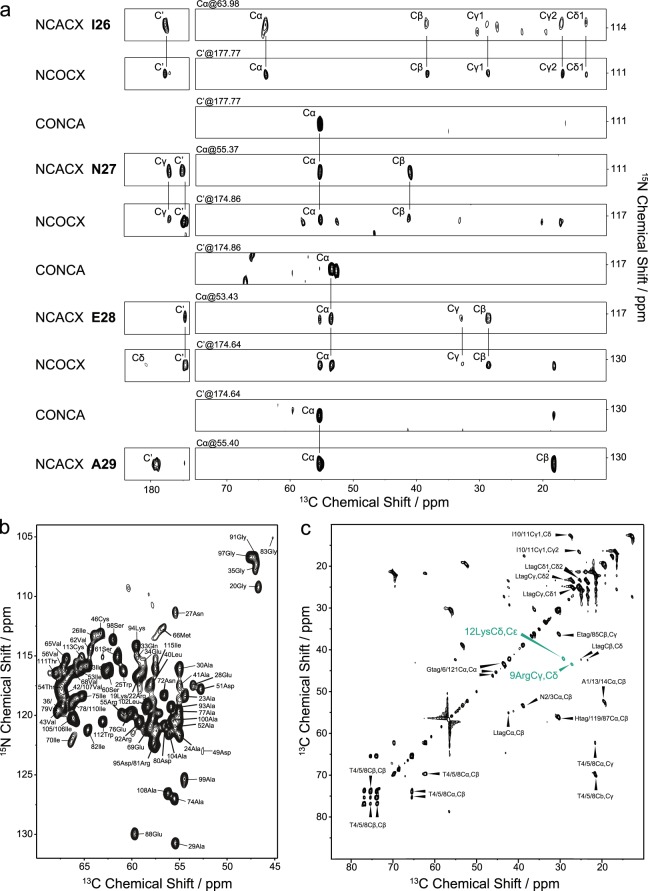
Spectra used for resonance assignment of DGK by MAS NMR. (**a**) Representative sequential assignment of U-^13^C,^15^N-DGK from I26 to A29 based on a set of dipolar-coupling based 3D NCACX, NCOCX and CONCA spectra. Each set of three spectra stands for a Cx[i-1]-N[i]-Cx[i] spin system. For example, the N27 NCACX peaks are linked to the I26-N27 CONCA peak through the same N and Cα and the I26 NCOCX peaks are connected to the I26-N27 CONCA peak *via* the same N and C’, thus generating a Cx[i-1]-N[i]-Cx[i] system. It is associated with the prior system through all carbon shifts of I26 that are visible in both NCACX and NCOCX spectra. All detailed experimental NMR parameters are summarized in Suppl. Table 4. (**b**) 2D NCA spectrum of U-^13^C,^15^N-DGK with all assigned peaks labelled. (**c**) 2D scalar-coupling based ^13^C-^13^C TOBSY of U-^13^C,^15^N-DGK with tentative assignments. INEPT and TOBSY were used for ^1^H-^13^C heteronuclear polarization and ^13^C-^13^C homonuclear mixing, respectively. Peaks for Arg9 and Lys12 are highlighted green, since they could be assigned unambiguously. Amino acids that correspond to the His-tag are labelled by ‘tag’.

**Figure 3:**
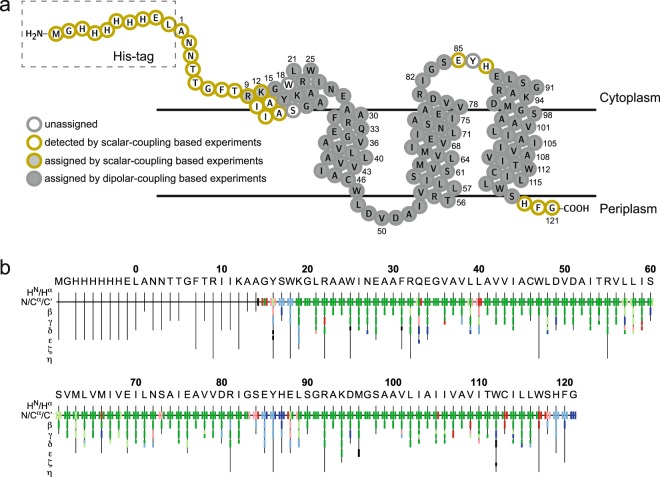
Resonance assignment of DGK. (**a**) Assigned residues mapped on the topology plot of DGK. The plot was created based on the DGK X-ray structure (PDB 3ZE5)^10^ and refined by CSI values obtained from chemical shifts (see Suppl. Fig. 6). 84% residues of DGK could be assigned by dipolar- and scalar-coupling based experiments. (**b**) Automated resonance assignment by ssFLYA confirms 91.5**%** of the backbone and 89.1**%** of all (backbone + sidechains) assignments obtained manually. Color code: green - assignment by ssFLYA agrees with the manual reference assignment within a tolerance of 0.55 ppm; red - assignment does not match with the reference; blue - assigned by ssFLYA, but not manually; black - assigned manually, but not by ssFLYA. Backbone and sidechain nuclei are shown. For branched sidechains, the relevant row is subdivided into an upper part for one branch and a lower part for the other branch.

We carried out sequential assignment of wild-type DGK using a combination of dipolar-coupling-based 3D experiments (NCACX, NCOCX, CONCA). The NMR time could be reduced by paramagnetic doping with Gd^3+^-DOTA^43^ in combination with a custom-built E-free probehead optimized for an increased duty cycle, which enabled using a recycle delay of just 0.8 s. Thereby, experiments could be recorded 3-times faster. Due to the high quality of the NMR spectra, we obtained ^13^C and ^15^N assignments using mainly uniformly labelled samples (U-^13^C,^15^N-DGK). The resolved ^15^N and ^13^C signals in 2D NCA spectra show average linewidths of 105 and 185 Hz, respectively. Residual ambiguities could be resolved by reverse labelling of isoleucine, leucine and valine (U-^13^C,^15^N-DGK-I,L,V) (Suppl. Fig. 4a). A representative sequential walk linking I26 to A29 *via* 3D NCACX, NCOCX and CONCA spectra is shown in Fig. 2a. We were able to assign the backbone and most sidechain carbon and nitrogen resonances for approximately 82% of the residues (=99 residues), from which 73 residues (~74% of the assigned residues) are completely assigned. Assigned resonances are highlighted in a 2D NCA spectrum (Fig. 2b) and mapped on the topology plot of DGK (Fig. 3a). For validation, we also applied ssFLYA, a generally applicable algorithm for the automatic assignment of protein solid-state NMR spectra^38^. So far, it has been used for microcrystals and amyloids^38^. Here, we tested its principal applicability for membrane proteins. Overall, 91.5% of the backbone and 89.1% of all (backbone + sidechains) assignments could be confirmed, verifying the manually obtained data set. The few disagreements occurred mainly in flexible regions with residues featuring smaller peak intensities or originated from spectral artefacts or missing peaks due to spectral overlap. A full overview of the automatic assignment is provided in Fig. 3b. As not all residues could be detected in cross-polarization (CP)-based experiments, scalar-coupling based spectra were also recorded. Similar to the data reported for the thermostable DGK mutant^44^, a number of mobile residues could so be observed. We recorded ^1^H-^15^N HETCOR, ^1^H-^13^C HETCOR and ^13^C-^13^C TOBSY spectra (Fig. 2c, Suppl. Fig. 5a–c) showing well-resolved peaks of “solution-state”-like quality due to motions on the submicrosecond time scale. Most of the peaks could be assigned to types of amino acids based on the BMRB database^45^, such as Ala, Arg, Asn, Gly, His, Ile, Leu, Lys, Phe and Thr. Thus, these peaks could be tentatively assigned to the two termini and the cytosolic loop between helix 2 and 3, where residues are located that are not detectable by dipolar-coupling based experiments. Peaks for arginine and lysine could be assigned unambiguously to Arg9 and Lys12 by exclusion, since all other arginines and lysines were already assigned by dipolar-coupling based experiments.

With careful optimization of the sample preparation and using the NMR strategy described above, we were able to assign 84% of the residues (=101 residues) located in transmembrane and extramembranous regions by dipolar- and scalar-coupling-based experiments. All assigned residues are mapped on the topology of DGK (Fig. 3a) and listed in the Suppl. Table 1. Residues of the termini and the cytosolic loop were not or only tentatively assigned, because they were too mobile for dipolar- but not mobile enough for scalar-coupling-based transfer experiments.

Although asymmetries were observed in the DGK X-ray structure^10^, no systematic peak doublets or triplets were detected during the assignment procedure. This is illustrated by a number of well-resolved peaks, such as Gly20, Asn27, Ala29, Glu88, Ala99 and Trp112 in the 2D NCA spectrum (Fig. 2b). Thus, it can be concluded that the DGK trimer adopts a symmetric conformation in liposomes.

Based on the resonance assignment, a secondary structure analysis was carried out using the chemical shift index (CSI)^46^. Suppl. Fig. 6 illustrates the comparison of the secondary structures of wild-type DGK, its thermostable mutant^29^ determined both by MAS NMR and the crystal structure of wtDGK (PDB 3ZE4)^10^. All three structures feature substantial similarities, especially concerning the high α-helical content. However, there are a few differences. In contrast to both MAS NMR secondary structures, the crystal structure shows small deviations around the flexible regions (see Suppl. Information for further details). The largest discrepancy concerns the length and position of the cytosolic loop (CL). It is shifted from residues 83–87 in the MAS NMR structure of wtDGK to residues 83–90 (subunit A), 86–91 (subunit B) and 82–87 (subunit C) of the X-ray structure.

### Functional studies by chemical shift perturbations

The chemical shift assignment allows probing the effects of nucleotide- and lipid substrate binding. We therefore compare the apo state of DGK with the nucleotide-bound state by using AMP-PCP, a non-hydrolysable ATP analogue. The DAG-bound state is probed by adding DOG, a slightly shorter DAG analogue. Perturbations in peak position and intensity of the substrate-bound states were analysed for each of the 101 assigned residues. Substrate-induced chemical shift perturbations indicate structural changes while alterations in peak intensities can be interpreted qualitatively in terms of altered dynamics: An increase in mobility causes a reduction in peak intensities in experiments based on cross polarisation.

#### The nucleotide-bound state of DGK

**Figure 4:**
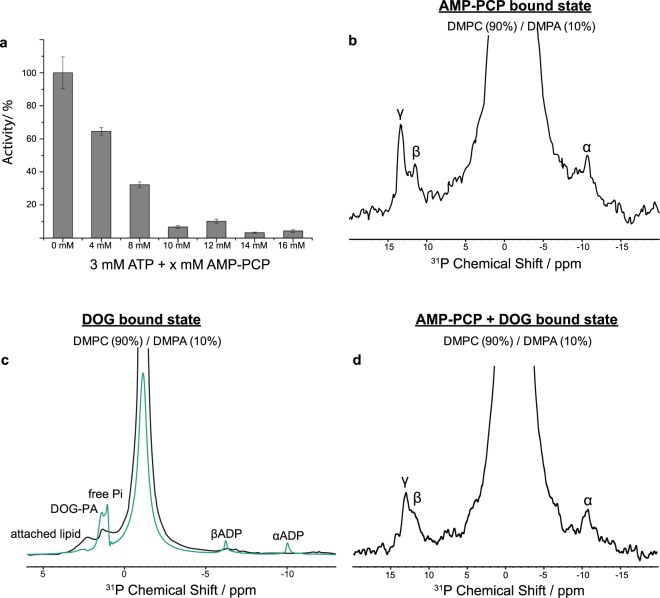
Trapping DGK in nucleotide- and/or lipid-bound states. (**a**) Competitive inhibition assay proves the binding of Mg*AMP-PCP to the active sites of DGK. DGK proteoliposomes were incubated with 4 to 16 mM of Mg*AMP-PCP, which equates 4 to 16- fold molar excess compared to DGK. A 3-fold molar excess of Mg*ATP (3 mM) compared to DGK was present in each sample. A concentration of at least 10 mM of Mg*AMP-PCP (10-fold molar excess) is necessary to reduce the activity of DGK below 10**%**, leading to a fully saturated system. 100% activity corresponds to the rate recorded with wtDGK in 90 mol% DMPC/10 mol% DMPA of 90 (±9.9) µmol min^−1^ mg^−1^. Experiments were repeated three times. The activity was calculated as the mean value. Error bars correspond to standard deviations. (**b**) Binding of AMP-PCP is confirmed by ^31^P-CP MAS. Proteoliposomes were incubated with a 14-fold molar excess of Mg*AMP-PCP (pH 7.2). (**c**) DGK was reconstituted into 80 mol% DMPC/DMPA and 20 mol% DOG and incubated with a 14-fold molar excess of Mg*ATP (pH 7.2). DGK phosphorylates DOG to DOG-PA, which can be detected by ^31^P-MAS NMR, both by cross- and direct polarization (in green) as described before^21^. Both nucleotide and DOG-PA have bound and non-bound populations. The CP experiment emphasizes the bound fraction. The spectra verify that DOG can reach the active site of DGK in our preparations. (**d**) ^31^P-CP spectrum of DGK reconstituted into 80 mol% DMPC/DMPA and 20 mol% DOG and incubated with a 14-fold molar excess of Mg*AMP-PCP (pH 7.2), verifying a binding of AMP-PCP to DGK by showing ^31^P species of the bound AMP-PCP.

In order to trap DGK in a stable nucleotide-bound state, a non-hydrolysable ATP analogue is required. Previous studies have shown that trapping with ADP.Vi only uncouples the phosphoryl transfer reaction, whereas ATPγS gets fully consumed^21^. In contrast, adenylylmethylenediphosphonate (AMP-PCP) was reported as a suitable analogue^11^. For finding saturation conditions, a competitive Mg*ATP inhibition assay was performed by monitoring the ATPase activity as a function of Mg*AMP-PCP concentration (Fig. 4a). At least a 10-fold molar excess of Mg*AMP-PCP compared to DGK is necessary to reduce its activity below 10%, leading to saturation. For all NMR experiments of DGK bound with Mg*AMP-PCP, DGK proteoliposomes were incubated with the nucleotide analogue at a molar ratio of 1:14. Using ^1^H-^31^P cross polarization, AMP-PCP bound to DGK could be detected under these conditions (Fig. 4b). We then verified that the fully saturated system is stable over at least 30 d at approximately 4 °C (Suppl. Fig. 4b), enabling the acqusition of large data sets such as 3D NCACX spectra with measurement times of up to 14 d.

**Figure 5:**
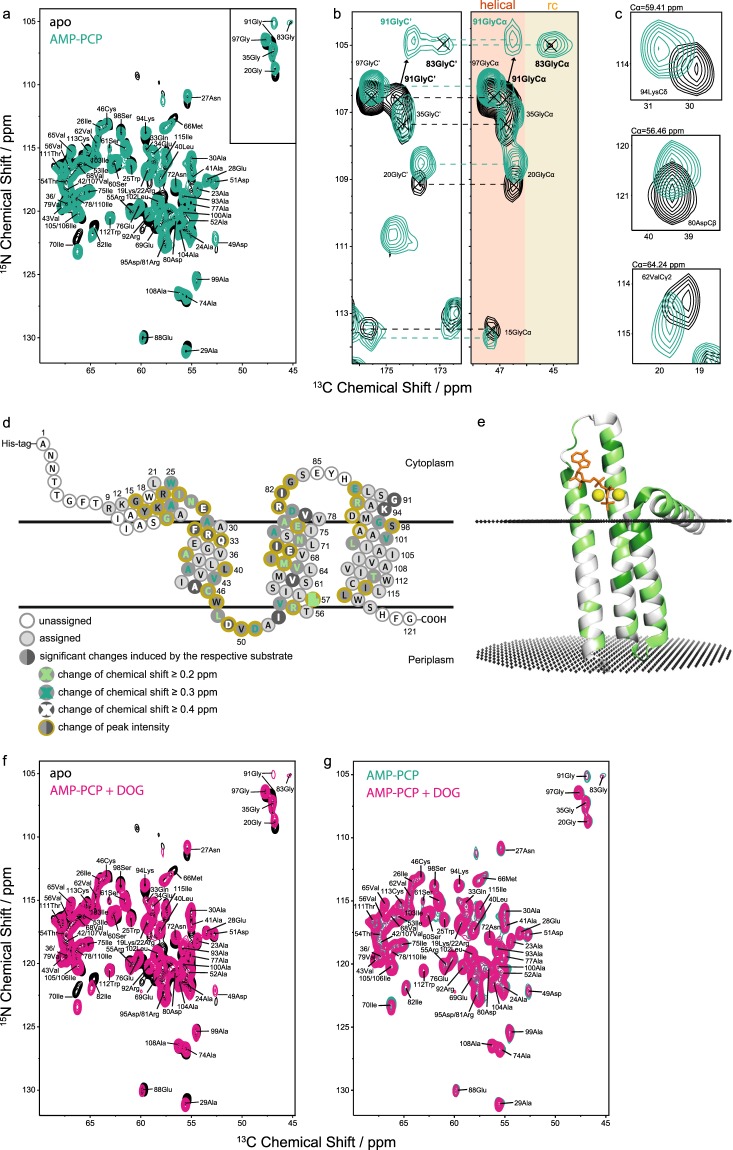
Effect of nucleotide binding on the MAS NMR spectra of DGK. (**a**) Superposition of 2D NCA spectra of apo DGK (black) and AMP-PCP-bound DGK (green). Representative pronounced shifts are shown in subsections of 2D NCACX (**b**) and 3D NCACX (**c**) spectra. In (**b**) regions for helical and random coil (rc) secondary structure are highlighted. (**d**) The topology of the DGK monomer is illustrated with residues highlighted that are affected by AMP-PCP. Changes in peak intensity and different levels of weighted CSPs are distinguished. All CSPs ≥0.2 ppm are counted as significant due to the high quality of the NMR spectra. The resolved ^15^N and ^13^C signals in 2D NCA spectra show average linewidths of 105 and 185 Hz, respectively. (**e**) The ribbon model of monomeric DGK shows residues, which are affected by AMP-PCP in green. It is obtained from the OPM database^72^, using the PDB ID 4UXX from the X-ray structure^11^. (**f**) Superposition of 2D NCA spectra of apo DGK (black) and AMP-PCP + DOG bound DGK (pink). (**g**) Superposition of 2D NCA spectra of AMP-PCP-bound (green) and AMP-PCP + DOG-bound (pink) DGK. Both the AMP-PCP bound and AMP-PCP + DOG bound states feature a similar fingerprint with significant alterations compared to the apo state. The observed changes are summarized in Suppl. Table 2.

As for the apo state, no peak splitting could be detected in the presence of AMP-PCP during the assignment process under the applied experimental conditions. This is already partially visible in the 2D NCA and 2D NCACX spectra, e.g. for Gly20, Asn27, Ala29, Glu88, Ala99 and Trp112 (Fig. 5a–c) and is indicative for a symmetric conformation of DGK in both states. The DGK X-ray structure^10^ shows asymmetries for example for Gly83 and Gly91. Both residues, framing the cytosolic loop involved in nucleotide binding^11^, should be sensitive to conformational asymmetries induced by AMP-PCP. However, no peak multiplets were observed upon AMP-PCP binding (Fig. 5b). Only one crosspeak for Gly83, implying a random coil structure and one peak for Gly91, suggesting a helical structure (based on CSI), could be detected. These findings support our assumption that the DGK trimer adopts a symmetric conformation not only in the apo- but also in the AMP-PCP bound state.

In the AMP-PCP bound state, significant chemical shift perturbations (CSPs ≥ 0.2 ppm) and effects on peak intensities are observable for 57% of the assigned residues, including backbone and sidechains (Fig. 5a–c, Suppl. Table 2). All CSPs ≥ 0.2 ppm are counted as significant due to the high quality of the NMR spectra. The resolved ^15^N and ^13^C signals in 2D NCA spectra show average linewidths of 105 and 185 Hz, respectively. Alterations of the peak intensities are defined as relevant, if an increase/decrease of at least 20% was seen. Previous mutational studies have identified the following residues of DGK to be functionally important: T8, R9, A13, S17, **G20, E28**, A30, **F31, R32**, E34, **E69, N72**, S73, **E76, D80, R81, G83**, L89, S90, A93, **K94, D95, G97, S98** and A100^12,16^. With our studies by MAS NMR using heteronuclear 2D and 3D correlation experiments, we were able to confirm 14 out of these 25 residues to be affected by nucleotide binding, showing significant CSPs and/or changes in peak intensities (labelled bold). Additionally, we could also identify further 43 residues: G15, Y16, K19, R22, A23, W25, I26, N27, A29, Q33, A37, L40, A41, V43, A45, C46, W47, L48, D49, V50, D51, I53, R55, V56, L57, V62, V65, M66, I67, I70, A74, A77, V79, I82, E88, G91, R92, A99, V101, L102, T111, I114 and L116. Affected residues, featuring clear CSPs and/or perturbations in peak intensity, are highlighted in the DGK topology plot and mapped onto the 3D structure in Fig. 5d,e. The picture shows that binding of the nucleotide has a more extensive impact on DGK as it was assumed so far^12^. Here, large CSPs ≥0.4 ppm are observable mainly for residues of the cytosolic region: E28, F31, R32, Q33, E69, I70, V79, R81, I82, G91 and K94, but also for the periplasmic loop: D49, I53 and the transmembrane region: A45, V62. For V62, D80 and K94, representative sections from the 3D NCACX spectra are shown in Fig. 5c. Especially glycines turned out to be sensitive to AMP-PCP binding. We could observe significant chemical shift changes for Gly15, Gly20, Gly91 and Gly97 and increased peak intensities for Gly20 and Gly83 (Fig. 5a,b). Particularly, Gly83, located in the cytosolic loop (CL), features a remarkable increase in signal intensity, suggesting a reduced mobility of CL. This is in agreement with X-ray data, indicating a participation of the cytosolic loop in nucleotide binding *via* Glu85, Tyr86 and His87^11^. However, the most pronounced effect can be seen for Gly91, located in the extramembranous part of helix 3 with a decrease of the N chemical shift by 1.7 ppm. A reason could be the close proximity to the purine ring of AMP-PCP^11^, leading to a higher shielding of Gly91 N. Overall, glycines serve as perfect probes for sensing nucleotide binding and accompanied conformational and dynamical changes in the cytoplasmic region.

#### DGK in the presence of lipid substrate

For generating a state of DGK with bound lipid substrate, we reconstituted the protein into DMPC/DMPA liposomes, including 20 mol% *sn*-1,2-dioctanoylglycerol (DOG, chain length n = 8), which has been reported as a suitable lipid substrate for DGK^21,47^. Empirically, it turned out that 20 mol% of DOG is an appropriate amount to keep the liposomes still intact. Under these conditions, DOG is in 10-fold excess over DGK. In order to test, whether DOG can reach the active site, MgATP was added to the sample so that DGK could catalyse the phosphoryl transfer reaction from ATP to DOG to yield DOG-PA. The latter should then become visible in ^31^P spectra. Indeed, the DOG-PA signal is observed in ^31^P-cross polarization (CP) and direct polarization (DP) spectra (Fig. 4c). The DP spectrum shows all ^31^P-species such as α- and β-ADP as well as DOG-PA. The CP spectrum on the other hand only displays membrane-bound ^31^P-species such as DOG-PA and phospholipids. Both the DP and CP spectra verify that DOG reaches the active sites of DGK in our preparations. The same conditions were used to prepare a sample, in which DGK was saturated with both Mg*AMP-PCP and DOG (80 mol% DMPC/DMPA and 20 mol% DOG). Of course, DOG could not be phosphorylated in this case but bound Mg*AMP-PCP could be detected as described above (Fig. 4d).

In the presence of DOG, overall alterations are clearly less pronounced than in the AMP-PCP bound state (see Suppl. Fig. S7 and Fig. 5f). The observed subtle effects involving mainly changes in peak intensities could be caused by specific direct response on the lipid substrate in the active site, but could also arise from changes of the membrane properties due to 20 mol% of DOG inserted into the lipid bilayer. It is reported that short chain DAGs as DOG (chain length n = 8) induce relatively small bilayer perturbation^48^. As for AMP-PCP, no peak splittings could be detected supporting a symmetric structure of DGK under these conditions. The AMP-PCP + DOG bound state features a similar fingerprint compared to the state with only AMP-PCP bound (Fig. 5f,g).

### Functionally relevant interprotomer interactions within the DGK trimer

DGK has been demonstrated to be highly stable in native membranes^13,14^. Additionally, the oligomeric arrangement of DGK is of direct functional relevance as the three active sites are formed by two adjacent protomers. We therefore tried to find specific intra- and interprotomer contacts in apo-state DGK, which are of structural or functional importance. We used both magnetization transfer experiments between sidechains using the experimental conditions described above and DNP-enhanced solid-state NMR on mixed-labelled DGK trimers. Here, interactions of interest are especially salt bridges such as those formed between Lys/Arg and Asp/Glu or H-bonds between sidechains as i.e. for Arg and Asn.

**Figure 6:**
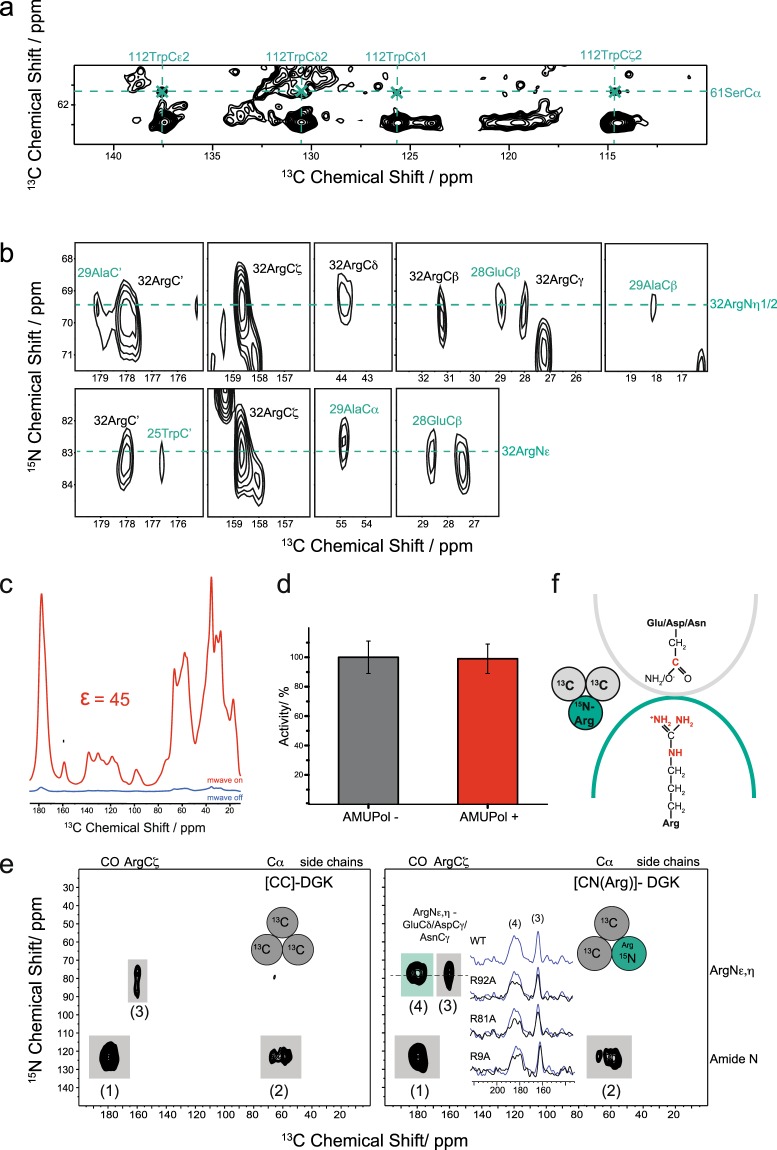
Intra- and interprotomer interactions in DGK. (**a**) 2D ^13^C-^13^C DARR spectrum of U-^13^C,^15^N-DGK-ILV with 800 ms mixing time. Crosspeaks occur between Cα of Ser61 and the sidechain carbons of Trp112, which demonstrates an intraprotomer contact between helices 2 (Ser61) and 3 (Trp112). (**b**) 2D NCOCX spectrum of U-^13^C,^15^N-DGK with a 400 ms DARR mixing step. Crosspeaks between 32ArgNη1/2 and 32ArgNε with 25TrpC’, Glu28Cβ and 29AlaCα/Cβ/C’ are detected, caused by an intraprotomer contact between these residues in helix 1 and the surface helix. (**c**) DNP enhancement illustrated for a ^13^C-CP spectrum of DGK incubated with 20 mM AMUPol. Upon microwave irradiation, a 45-fold sensitivity enhancement is gained. (**d**) Activity of DGK with (+) and without (−) AMUPol, demonstrating that the presence of the biradical has no influence on the activity. 100% activity corresponds to the rate recorded with wtDGK in 90 mol% DMPC/10 mol% DMPA of 90 (±9.9) µmol min^−1^ mg^−1^. Experiments were repeated three times. The activity was calculated as the mean value. Error bars correspond to standard deviations. (**e**) DNP-enhanced ^15^N-^13^C-TEDOR spectra of mixed-labelled [CN(Arg)]-DGK trimers (right) and of a U-^13^C-labelled control sample ([CC]-DGK) (left). Both spectra show natural abundance crosspeaks (grey box): intra-protomer N-C’ (peak 1), intra-protomer N-Cα (peak 2), intra-residue Arg-Nε,η-Arg-Cζ (peak 3). An additional crosspeak (green box) is observed in [CN(Arg)]-DGK (peak 4). It can be assigned to an interprotomer contact, representing a through-space correlation between Arg and Asn/Asp/Glu. (**f**) This cross-peak demonstrates that salt bridges or H-bonds between Asp/Glu/Asn and Arg must exist at the protomer interfaces. Mutations R9A, R81A and R92A cause a reduction in intensity of peak (4) (inset in (**e**)) demonstrating their involvement in these cross-protomer contacts (see Fig. S8d for further details). The TEDOR spectra were recorded with a mixing time of 6.25 ms.

**Figure 7:**
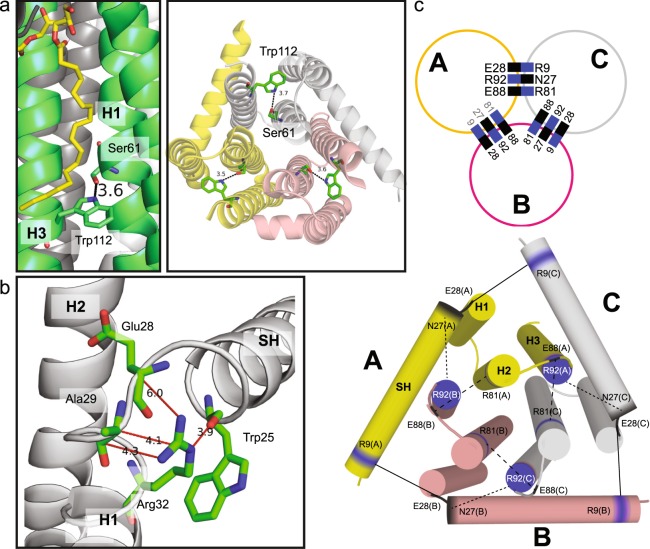
Intra- and interprotomer interactions in DGK visualized in the crystal structure of Δ4 DGK (PDB 4UXX)^11^ (**a**) or (PDB 3ZE5)^10^ (**b**). (**a**) The intraprotomer contact between Trp112 and Ser61 within one monomer viewed from the membrane plane (left, lipid substrate in yellow) and from the periplasm (right). The different monomers are shown in pink, yellow and grey, respectively. Trp112 (H3) forms a hydrogen bond with Ser61 (H2) in the lower part of the hydrophobic pocket. (**b**) Enlarged view from the membrane plane, showing the intraprotomer contacts between the guanidino group of 32Arg with 25TrpC’, 28GluCβ and 29AlaCα/β/C’. (**c**) Model for the cross-protomer interactions in DGK involving Arg9 (solid line), 81 (large dashes) and 92 (small dashes), viewed from the cytoplasm. Potential interaction partners are proposed based on their location as deduced from available crystal structures (3ZE4, 3ZE5, 4UXX). SH: surface helix, H1/2/3: transmembrane helices 1/2/3.

First, utilizing our extensive sidechain assignment, ^13^C-^13^C DARR and 2D NCOCX spectra with long mixing times were recorded for apo state DGK. The spectra were searched for long-range, non-sequential sidechain-sidechain crosspeaks. In this way, some intraprotomer contacts were found: Crosspeaks between Cα of Ser61 and sidechain carbons of Trp112 (Fig. 6a) demonstrate close proximity of both residues. Trp112 is located in the transmembrane part of helix 3 and Ser61 in helix 2, which is in agreement with findings of Caffrey and co-workers, who assumed a hydrogen bond between Nε of Trp112 and the OH-group of Ser61 (Fig. 7a)^11^. Interestingly, crosspeaks between sidechain nitrogens, Nε and Nη1/2, of Arg32 with 25TrpC’, 28GluCβ and 29AlaCα,Cβ,C’ are found (Fig. 6b). Arg32 is located at the membrane/cytoplasm interface of helix 1, while Trp25, Glu28 and Ala29 are found in the SH, interhelical turn and the cytoplasmic part of H1, respectively (Fig. 7b). This contact has not been described before. In order to test its functional relevance, we have prepared a R32A mutant, which shows a strongly reduced activity (Suppl. Fig. 8c).

Since no cross-protomer contacts could be detected in this way, mixed-labelled DGK trimers were prepared (Suppl. Fig. 8a). In such samples, interactions across the oligomerisation interfaces could be monitored by dipolar through-space N-C correlation spectroscopy as demonstrated by Maciejko *et al*.^36^. As such contacts are expected to be sparse and weak, sensitivity enhancement by dynamic-nuclear-polarization (DNP) is essential^36^. Here, a signal enhancement of 45 was achieved for U-^13^C-DGK by using the stable and soluble biradical AMUPol^49^ as a polarizing agent (Fig. 6c). DNP creates proton polarization in direct proximity to the biradical and gets evenly distributed through the sample via the homogeneously coupled proton network by spin diffusion at a temperature of 100 K. Transfer from protons to carbons and nitrogens takes then place by cross polarization. The presence of AMUPol has no influence on the activity of DGK (Fig. 6d).

Here, a labelling scheme was selected, in which U-^13^C-DGK was mixed with ^15^N(Arg,Lys)-DGK. In the resulting samples ([CN(Arg,Lys)]-DGK), cross-protomer salt bridges or H-bonds (Arg/Lys-Glu/Asp/Asn) can be detected by DNP-enhanced N-C TEDOR experiments. A statistical analysis of salt bridges within proteins reveals distances between Nη1/2 and CO in the corresponding sidechain of around 3 Å^50^. We have also measured such a distance for a cross-protomer salt bridge within a membrane protein complex by TEDOR in one of our previous papers^36^. We have therefore selected a TEDOR mixing time (6.25 ms) which provides largest peak intensities for these distances.

N-C TEDOR spectra of a mixed and selectively labelled [CN(Arg,Lys)]-DGK and of ^13^C-only labelled [CC]-DGK are shown in Fig. 6e. Both spectra reveal natural abundance signals originating from N-C’ (crosspeak (1)) and N-Cα (crosspeak (2)) single bond contacts. Additionally, arginine intraresidue ^13^C-^15^N contacts between ArgNε,η and ArgCζ can be observed (crosspeak (3)). One additional resonance (crosspeak (4)) is only observed in the mixed labelled [CN(Arg,Lys)]-DGK, which must therefore arise from cross-protomer contacts.

This crosspeak represents a through-space correlation between a ^15^N resonance of Arg-Nε,η with a ^13^C resonance of a carboxyl group from Asp-Cγ and/or Glu-Cδ and/or the carbonyl group from Asn-Cγ. It therefore demonstrates the occurance of one or more salt bridges or H-bonds between DGK protomers (Fig. 6f). In order to assign which of the 6 Arg residues in DGK contributes to these interactions, single-site RxA mutations were introduced and mixed-labelled complexes were prepared (Fig. S8b,c). The resulting TEDOR spectra show a clear intensity reduction of crosspeak (4) upon introducing mutations R9A, R81A and R92A (see insert in Fig. 6e). In contrast, mutations R22A, R32A and R55A had no significant effect (Fig. S8d). This means that R9, R81 and R92 are contributing to cross-protomer interactions and therefore to crosspeak (4) observed for the wildtype in Fig. 6e.

## Discussion

We have assigned the chemical shifts of wild-type DGK within a lipid bilayer by 3D MAS NMR. This data set enabled us to probe the response of DGK towards nucleotide and lipid substrate binding. Furthermore, conventional as well as DNP-enhanced MAS NMR was applied to resolve functionally important long-range contacts. We found clear evidence that the DGK trimer adopts a symmetric conformation in its apo state. Previously reported X-ray structures of DGK featured asymmetries in the secondary structure between the three subunits^10,29^. The differences between NMR and X-ray data in conformational symmetry of apo-state DGK might arise from different experimental conditions. Especially crystal packing could be a potential source for structural asymmetries^51^. In general, high dynamics of the loop regions, which are reflected by higher B-factors and chain displacements^10^, may lead to ambiguities in defining loop positions and lengths.

Under the applied experimental conditions, the DGK trimer adopts a symmetric conformation in the presence of AMP-PCP and DOG. This indicates that all three active sites are occupied by the substrate at the same time. Thus, our data imply that under high substrate concentration, the three active sites are most likely simultaneously in the same state during catalysis, which differs from the proposal by Caffrey and co-workers^11^. The crystal structure of Δ4DGK co-crystallized with AMP-PCP shows that only one active site is occupied by the nucleotide substrate, although AMP-PCP was used at high concentration (10 mM)^11^. Differences in the number of occupied active sites between NMR and X-ray data might arise from different experimental conditions. Our investigations by MAS ssNMR were carried out on wild-type DGK in synthetic lipid bilayers at pH of 7.2 and 275 K. Crystallization of the thermostable Δ4 mutant took place under acidic conditions in lipidic cubic phases formed by monoolein, a DGK substrate. Its structure could be slightly affected by crystallization contacts. Since the assignment covers nearly the entire protein, we had a very good basis for mapping changes during substrate binding.

For the AMP-PCP bound state, we could observe for the first time that not only the cytosolic part but also large parts of the transmembrane domains are affected by nucleotide binding (Fig. 5d,e). The conformational change induced by the nucleotide, most likely directing DGK into its catalytic active form, is in good agreement with the observation that high nucleotide specificity of DGK is mainly seen in form of reductions in k_cat_ for ATP analogues^17^. It is also supported by the fact that the tetraphosphate-linked bisubstrate analogue acted as a good inhibitor^17^.

Additionally, it is not surprising that nucleotide binding causes so many chemical shift perturbations in such a small kinase. With respect to the size of DGK, the nucleotide is comparatively bulky and under our experimental conditions, three nucleotide molecules seem to bind per trimer. In such case, the membrane becomes an important stabilizing factor as pointed out by Jia and co-workers^27^. A similar substantial conformational change was observed for other kinases as well, which also catalyse direct phosphoryl transfer^52–54^. Accordingly, binding of nucleotide induces an information transfer through the whole enzyme towards the opposite site, which might occur in preparation of DGK for binding the lipid substrate. The respective residues are possibly oriented for binding of the lipid. Consequently, we propose positive heteroallostery, emanating from the nucleotide substrate, which is in agreement with kinetic studies of DGK, indicating that binding of the nucleotide substrate does result in an enhanced affinity for the lipid substrate^9,17^.

For the bound states of DGK with either AMP-PCP + DOG or only AMP-PCP, a similar fingerprint was observed (Fig. 5f,g). This indicates that the nucleotide substrate induces a substantial conformational change, which might be necessary to trigger the actual phosphoryl transfer reaction. Kinetic and structural data support a direct, in-line phosphoryl transfer based on a close proximity of the γ-phosphate of the nucleotide and the 1-OH of the lipid substrate^11,17,21^. Respectively, our data suggest that the specific conformation induced by the nucleotide is most likely DGK’s catalytic active conformation, which is necessary to bring both substrates in close proximity.

As a control, the effect of different lipid compositions on the spectral fingerprint and activity of DGK has been probed. Kinetic studies by Lee and co-workers indicated that product analogues of DGK’s physiological forward reaction, such as DMPA, possibly bind to the active site^55^. Pilot *et al*. demonstrated that 1,2-dioleoyl-sn-glycero-3-phosphate (DOPA) increases the Km of the lipid substrate 1,2-dihexanoylglycerol (DHG). This has been explained by a possible binding of the product analogue to the active site in competition with the lipid substrate. However, the superposition of the 2D ^13^C-^13^C PDSD spectra of DGK embedded into 90 mol% DMPC/10 mol% DMPA, 100 mol% DMPC and 90 mol% DMPC/10 mol% DMPG (Suppl. Fig. 3a–c) shows the same fingerprint for DGK in all three liposome compositions with no chemical shift perturbations observable. Additionally, DGK features a similar activity in all three liposome compositions (Suppl. Fig. 3d). This indicates that under our experimental conditions DMPA does not seem to bind to DGK’s active site, which in turn suggests that DGK exists in an apo state when it is reconstituted into 90 mol% DMPC/10 mol% DMPA. This observation differs from the previous kinetic studies but could be explained by the different experimental conditions. For the kinetic studies by Lee and co-workers, DGK was reconstituted into 80 mol% DOPC/20 mol% DOPA in a molar lipid-to-protein ratio of 6000:1, which results in a remarkable high molar PA-to-protein ratio of 1200:1. In our study by MAS NMR, DGK was reconstituted into 90 mol% DMPC/10 mol% DMPA in a much lower molar lipid-to-protein ratio of 50:1. This implies that a very high molar excess of PA compared to DGK seems to be necessary to force its binding to the enzyme. In addition, Lee and co-workers carried out the activity measurements on unsealed membrane fragments consisting of phospholipids and detergent. In contrast, DGK was present in detergent-free liposomes during the MAS NMR experiments presented here.

Furthermore, we were able to detect intraprotomer, long-range contacts by conventional high field ssNMR. The contact between Trp112 and Ser61 (Fig. 6a) has possibly a stabilizing effect on the transmembrane region of each monomer (Fig. 7a). This is consistent with mutational studies by Lau and Bowie, who found that the mutant of DGK, in which Trp112 is replaced by Phe, was prone to aggregation and featured a low specific activity^14^. The contacts between Arg32Nε/Nη1, 2 and 25TrpC’, 28GluCβ and 29AlaCα,β (Fig. 6b) have most likely a strengthening effect on the joint between H1 and SH, stabilizing the SH and hence the active site as well (Fig. 7b). Arg32 could be shown to be functionally relevant (Suppl. Fig. 8c)^11,12^, which could be explained by its role in forming a contact, most likely providing a stabilization of the active site.

DNP-enhanced TEDOR spectroscopy on mixed-labelled DGK trimers and site-directed mutagenesis provided evidence for interprotomer contacts involving Arg9, Arg81 and Arg92. Unfortunately, our dataset does not allow an identification of their interaction partners. We have therefore analyzed the general topology and the available crystal structures of DGK in order to find Asp/Glu/Asn residues, which are located at suitable positions at the protomer interfaces. A likely interaction partner for Arg9 located at the beginning of SH of protomer A could be Glu28 at the end of the SH in the adjacent protomer B. The location of Arg81 at the cytoplasmic end of H2 in protomer A matches that of Glu88 in protomer B. Residue Arg92 at the cytoplasmic end of H3 in protomer A could interact with Asn27 in protomer C. The resulting interaction pattern, which is located at the cytoplasmic side of DGK, is illustrated in Fig. 7c. It is compatible with the overall topology of DGK. It is important to point out, that the exact sidechain orientations vary in between the available crystal structures and between protomer interfaces, which could be due to the described asymmetry as discussed above. However, cross-protomer N-C distances as close as 3.4–3.6 Å for 81Arg-Nη1,2/88GluCδ and 92Arg-Nη1,2/27AsnCγ can be found e.g. in structures 3ZE4, 3ZE5 and 4UXX^10,11^. Interactions of Arg9 are ambiguous as the surface helix is only partially resolved in these 3D structures. The Arg81-Glu88 interaction could stabilize the cytoplasmic loop (CL), which was reported to be involved in binding the nucleotide^11^. It is assumed that the Tyr86 sidechain of CL serves as a cover of the nucleotide binding site in the nucleotide bound state^10^. The Arg81-Glu88 contact might keep the cover open in the apo state to enable binding of the nucleotide. The interaction between Arg92-Asn27 links H3 and SH. Residue Asn27 is located at the end of the SH at the interhelical turn, which connects SH and H1^11^.The Arg92-Asn27 contact most likely keeps both the SH and the H1 closer to H2 and H3, stabilizing the active site. All three Arg could be shown to be functionally relevant by mutational studies (Suppl. Fig. 8c)^11,12^, although they are not reported to interact directly with the nucleotide or lipid substrate^11^. This observation supports their involvement in stabilizing the active site and/or providing a cross-talk between DGK protomers. However, it should be noted that all RxA mutations affected the DGK activity, regardless of their involvement in cross-protomer contacts.

## Conclusion

The application of high-field 3D-MAS NMR enabled us to obtain an almost complete resonance assignment of wtDGK within the phospholipid (DMPC/DMPA) bilayer in its apo- and its substrate-bound states. This data set allows mapping the overall response of DGK towards substrate binding. We could show that all three active sites can be occupied concurrently by AMP-PCP. Our data obtained under high substrate concentration demonstrate that the three active sites are in the same state during catalysis. Additionally, we could observe that not only the cytosolic part but also large parts of the transmembrane domains are affected by nucleotide binding. This most likely supports the enzyme in binding of the lipid substrate, indicating positive heteroallostery. Additionally, the substantial conformational change induced by the nucleotide seems to set the enzyme into a catalytically active state, triggering the actual phosphoryl transfer reaction. Furthermore, functionally relevant inter- and intraprotomer long-range interactions could be identified, which may stabilize the active sites and/or transmit information about substrate binding or changes of the surrounding lipid bilayer between protomers.

A number of technical aspects are worth noting. This study presents a combined use of high-field MAS NMR on uniformly labelled DGK proteoliposomes with DNP-enhanced MAS NMR on mixed-labelled DGK trimers. For assignment, highest possible resolution was necessary, which prevented the use of DNP. Instead, a moderate but still essential enhancement of data acquisition was possible by utilizing Gd-DOTA doping. Further improvements can be anticipated by combining proton-detected MAS NMR^56^, the use of optimum control pulse sequences^57^ and the application of non-uniform sampling^58^. The assignment process itself becomes faster and more reliable by automatic schemes such as ssFLYA^38^, as demonstrated here. For observing long-range cross-protomer contacts, signal enhancement by the use of DNP with specific labelling schemes was mandatory. The combination of both approaches allows obtaining valuable data on membrane proteins in the lipid bilayer, which are complementary to 3D structure determination by crystallization-based methods.

## Materials and Methods

### Sample preparation

#### Preparation of uniform and reverse labelled samples

Samples were basically prepared as described previously^21^: The synthetic gene coding for wild-type diacylglycerol kinase was cloned into the plasmid vector pSD005, a derivative of pTrcHisB. The encoded protein incorporates an N-terminal leader sequence containing a hexahistidine-tag for purification. The expression of uniformly labelled DGK took place in *E. coli* T7 express cells (New England BioLabs) using M9 minimal medium with [U-^13^C]glucose and [^15^N]ammonium chloride. For the preparation of reverse labelled samples, the amino acids Ile, Leu and Val were added to the medium to suppress isotope labelling of these residues. *E. coli* cells were grown until an OD_600_ = 0.6–0.8 was reached, whereupon protein expression was induced by the addition of 200 mg/L isopropyl β-D-1-thiogalactopyranoside (IPTG). Cells were harvested by centrifugation after 16 h of protein expression at 27 °C and 220 rpm.

The protein was solubilized in 3% (w/v) n-octyl-β-D-glucopyranoside (OG) (AppliChem). The solubilized protein (supernatant) was obtained by centrifugation at 10,000 rpm for 30 min and incubated with a Ni-NTA resin (Quiagen) at 4 °C for 1 h. Due to its N-terminal hexahistidine-tag, the protein binds to Ni-NTA. The bound protein was washed with 1.5% (w/v) OG and 50 mM imidazole. The detergent was exchanged by washing with 0.05% (w/v) n-dodecyl-β-D-maltoside (DDM) (AppliChem). The protein was finally eluted with 400 mM imidazole in 0.05% (w/v) DDM followed by the determination of its concentration by absorption spectroscopy at 280 nm on a UV-550 spectrophotometer (Jasco). The final yield of pure ^13^C, ^15^N labelled DGK was 30–45 mg/L. The purity was checked by SDS-PAGE, while the oligomeric state was analyzed by BN-PAGE (Life Technologies, NativePAGE Novex Bis-Tris) as described previously^36^.

For solid-state NMR experiments, DGK was reconstituted in DMPC/DMPA (90 mol%/10 mol%) liposomes with a molar protein/lipid ratio of 1:50. For this purpose, the protein was mixed with the liposomes and incubated for 1 h at 22 °C. Detergent removal was done with SM-2 BioBeads. BioBeads (80 mg/ml) were added and incubated overnight at 4 °C followed by 3 further additions every 2 h at 22 °C.

A sucrose gradient (40–10%) was carried out to verify a homogenous protein reconstitution into the liposomes. In order to reduce the NMR time, paramagnetic doping with 2 mM Gd^3+^-DOTA^43^ was carried out. The proteoliposomes were sedimented by ultracentrifugation and packed into a 3.2 thin wall MAS rotor. Approximately 18 mg of DGK could be loaded into the rotor.

#### Preparation of mixed labelled samples

For the detection of interprotomer contacts, mixed-labelled DGK was prepared. For the identification of the detected interprotomer Arg-contact, single-site RxA mutants were prepared. All single-site mutations were introduced to the wtDGK template vector by PCR amplification with overlapping mutagenic primers. Vectors were transformed into *E. coli* competent C43 (DE3) cells using the heat shock procedure and were subsequently plated on ampicillin-containing LB agar plates. Colonies were picked and grown in LB medium, and the plasmid DNA was isolated *via* an extraction kit. Sequences of wtDGK and all single-site mutant constructs were verified at Eurofins MWG Operon.

The expression and purification of mixed labelled DGK was in general performed as described above with the difference that ^13^C- and ^15^N-labelled samples were expressed separately to create mixed samples that only exhibit interprotomer and no intraprotomer ^13^C-^15^N contacts. For the selectively labelled sample, ^15^N-labelled arginine and lysine were added to M9 minimal medium. ^12^C-enriched glucose (99.5%) was used in ^15^N-labelled samples instead of normal glucose to suppress ^13^C natural abundance within a protomer. In order to disrupt DGK-trimers into monomers, the purified protein was incubated in SDS (0.2 mg/ml protein in 2% SDS) over night at RT. Here, the different labelled monomers were mixed in a 1:1 ratio. After the disruption and mixing procedure, SDS was exchanged by 0.5% DDM during a washing step to regain trimeric DGK. Then, the protein was reconstituted into 1,2-dimyristoyl-sn-glycero-3-phosphocholine (DMPC, 90 mol%)/1,2-dimyristoyl-sn-glycero-3-phosphate (DMPA, 10 mol%) liposomes with a molar protein/lipid ratio of 1:50 as described above. Using this approach, samples were prepared, in which DGK consists of ^13^C-DGK and ^15^N-DGK protomers ([CN]-DGK or [CN]-DGK-RxA) or ^13^C-DGK and ^15^N-Arg-Lys-DGK protomers ([CN(Arg,Lys)]-DGK) or ^13^C-DGK ([CC]-DGK). The latter is a control sample for analyzing ^13^C-^15^N contacts arising from naturally occurring ^13^C- or ^15^N-isotopes. The analysis of DGK was carried out by BN-PAGE (Life Technologies, NativePAGE Novex Bis-Tris) as described previously^36^ and the coupled activity assay (see below).

Reconstituted protein samples were doped with the polarizing agent AMUPol^49^ in order to achieve DNP signal enhancement. Two proteoliposome pellets, each ~20 µl, were covered with ∼20 μL of a 20 mM AMUPol solution (60% D_2_O, 30% glycerol-d8, 10% H_2_O) and incubated for 20 h at 4 °C. The solution was completely removed before the sample was packed into a 3.2 mm ZrO_2_ rotor.

#### Preparing substrate-bound states of DGK

In order to saturate DGK with nucleotide substrate, reconstituted DGK was incubated with a 14-fold molar excess of the ATP analogue adenylylmethylenediphosphonate (AMP-PCP) and a 28-fold molar excess of MgCl_2_ over night at 4 °C. Mg^2+^ is an essential catalyst for ATP hydrolysis. Additionally, Bell and co-workers could demonstrate that DGK requires next to Mg^2+^ complexed with ATP a free second divalent ion for activation with a preference for Mg^2+ ^^59^. AMP-PCP and MgCl_2_ were dissolved in 50 mM HEPES (pH 7.2). In order to saturate DGK with the lipid substrate, it was reconstituted into liposomes consisting of 80 mol% DMPC/DMPA (9:1) and 20 mol% *sn*-1,2-dioctanoylglycerol (DOG, n = 8). For the production of DGK saturated with both the nucleotide and the lipid substrate, it was firstly reconstituted into 80 mol% DMPC/DMPA (9:1) and 20 mol% DOG (n = 8) and then incubated with 14 mM AMP-PCP and 28 mM MgCl_2_ over night at 4 °C. All proteoliposome samples with the respective substrate(s) were doped with 2 mM Gd^3+^-DOTA, sedimented by ultracentrifugation and packed into a 3.2 thin wall rotor.

### Activity Assays

The activity of reconstituted DGK was determined at 30 °C using a coupled enzyme assay^17^ as described previously^21^. The activity was stimulated by the water-soluble lipid substrate analogue 1,2-dibutyrylglycerol (DBG). The synthesis of DBG will be reported elsewhere. The activities of all samples used here are summaraized in Suppl. Table 3.

### Solid-state NMR spectroscopy

Unless stated otherwise, all MAS NMR experiments presented here were carried out on a Bruker wide bore Avance III solid-state NMR spectrometer with a ^1^H frequency of 850.32 MHz. A sample spinning rate of 15.2 kHz was applied in each case. All samples were adjusted to a temperature of 275 K and pH 7.2. The NMR time of dipolar-coupling based experiments could be reduced by paramagnetic doping with Gd^3+^-DOTA^43^ in combination with an E-free 3.2 mm triple-resonance HCN MAS probehead, which enabled using a recycle delay of 0.8 s. Thereby, ~3x of the measurement time could be saved compared to the standard probehead (recycle delay of 2.5 s). The E-free probehead was custom-built by Bruker.

#### Manual resonance assignment

For the sequential assignment of the immobile domains of DGK, a combination of dipolar-coupling based 3D experiments (NCACX, NCOCX, CONCA)^60,61^ was carried out. Since we are able to observe well-resolved NMR-spectra of high signal-to-noise ratio, we mainly performed ^13^C and ^15^N assignments using uniformly labelled samples (U-^13^C,^15^N-DGK). Residual ambiguities were resolved by reverse labelling of isoleucine, leucine and valine (U-^13^C,^15^N-DGK-I,L,V). The experiments were either performed with an E-free or standard 3.2 mm triple-resonance HCN MAS probehead (Bruker). All experimental parameters are listed in Suppl. Table 4.

For the tentative assignment of highly mobile residues of DGK, scalar-coupling based 2D experiments (^13^C-^13^C TOBSY, ^1^H-^13^C HETCOR, ^1^H-^15^N HETCOR)^44,62,63^ were applied. Therefore, only the uniformly labelled sample (U-^13^C,^15^N-DGK) was used. The experiments were performed with a standard 3.2 mm triple-resonance HCN MAS probehead (Bruker). Typical 90° pulse lengths were 2.5 µs (^1^H), 4.5 µs (^13^C) and 6 µs (^15^N). A recycle delay of 2 s and a SPINAL64 ^1^H decoupling of 100 kHz were used. For all heteronuclear transfer steps a refocused INEPT (insensitive nuclei enhanced by polarization transfer)^64^ was applied with scalar couplings of 200 Hz (HC) and 90 Hz (HN). For the ^13^C-^13^C homonuclear polarization transfer, TOBSY (through-bond correlation spectroscopy)^63^ was applied with a 3.75 ms P9^1^_6_ mixing sequence.

#### Substrate-bound states

The binding of the substrate(s) to DGK was verified for each case (AMP-PCP, DOG + ATP, DOG + AMP-PCP) by ^31^P-CP (cross polarization) and ^31^P-DP (direct polarization) experiments. Therefore, the standard 3.2 mm double-resonance HX MAS probehead (Bruker) was used. Typical 90° pulse lengths were 3 µs (^1^H) and 4 µs (^31^P). A recycle delay of 3 s and a SPINAL64 ^1^H decoupling^65^ of 83.3 kHz were used. The CP contact time was 5 ms. All CP spectra were recorded with 16000 scans, while the DP spectra were carried out with 2000–4000 scans.

Scalar and dipolar coupling based experiments of DGK in its apo state, saturated with AMP-PCP, DOG and with AMP-PCP + DOG were carried out for comparison between the different states of DGK. The experiments were either performed with an E-free or standard 3.2 mm triple-resonance HCN MAS probehead (Bruker). All experimental parameters are listed in Suppl. Table 4.

#### Visualizing interprotomer contacts

DNP-enhanced MAS NMR spectra were recorded on a Bruker 400 DNP system consisting of a 400 MHz WB Avance II NMR spectrometer, a 263 GHz Gyrotron as microwave source, and a 3.2 mm HCN Cryo MAS probe. All experiments were conducted with 8 kHz MAS, and the microwave power at the probe was 12.5 W. During DNP experiments, the temperature was kept at 105 K. For all experiments, a SPINAL64 ^1^H decoupling^65^ of 100 kHz was applied during acquisition. A recycle delay of 2.2 s was used. 2D ^15^N-^13^C correlation spectra were acquired using the z-filtered TEDOR sequence^66^. Typical pulse lengths of 2.5 µs (^1^H 90°), 4.0 µs (^13^C 90°), 8.0 µs (^13^C 180°), 7.5 µs (^15^N 90°) and 15 µs (^15^N 180°) were applied. The CP contact time was 1.1 ms. A mixing time of 6.25 ms (24 rotor cycles) was used for all experiments. The 2D-spectra for [CC]DGK and [CN(Arg,Lys)]DGK were acquired with 1504 scans in the direct dimension and 60 increments of 125 µs each in the indirect dimension. The FID acquisition time in the direct dimension was 10 ms. The ^15^N pulse carrier was set to 54 ppm and the ^13^C pulse offset was set to 174 ppm. The 2D-spectra for [CN]DGK and [CN]DGK-RxA were acquired with 2880 scans in the direct dimension and 25 increments of 250 µs each in the indirect dimension. The FID acquisition time in the direct dimension was 10 ms. The ^15^N pulse carrier was set to 100 ppm and the ^13^C pulse offset was set to 174 ppm.

### Data analysis

All spectra were processed in TopSpin 3.5.b.91. pl 7 (Bruker). For comparison, the respective spectra were processed the same way. All multi-dimensional data were analyzed in CcpNmr Analysis 2.4.2^67^.

#### Chemical shift referencing

For the first 3D NCACX spectrum ^13^C chemical shift referencing was carried out with respect to DSS (4,4-dimethyl-4-silapentane-1-sulfonic acid) through the carbonyl of alanine (179.85 ppm). ^15^N chemical shifts were indirectly referenced to liquid NH_3_ at 0 ppm through the ^13^C/^15^N gyromagnetic ratio. All other spectra were subsequently referenced to an isolated resonance in this spectrum. ^31^P chemical shift referencing was performed with respect to 10% phosphoric acid at 0 ppm through crystalline triethylphosphine sulfide (TEPS) (58.62 ppm).

#### Automatic resonance assignment

In addition to manual resonance assignment, we applied the automatic resonance assignment algorithm for solid-state NMR spectra, ssFLYA^38^, in order to test its applicability to membrane proteins. It is based on the automatic assignment algorithm FLYA for solution NMR^68^. Both are implemented in the software package CYANA^69,70^. ssFLYA was applied as described previously^38^. As input, it used solely the sequence of DGK and the unassigned peak lists from dipolar-coupling based 3D experiments (NCACX, NCOCX, CONCA) obtained with the uniform (U-^13^C,^15^N-DGK) and reverse (U-^13^C,^15^N-DGK-I,L,V) labelled sample. The same manually prepared peak lists were used as for the manual assignment. Obvious artifacts were removed from the peak lists. The N-terminal region (-Met-9-Ala-14) of the protein sequence, which includes a His-tag is known to be highly mobile and not detectable in the NMR spectra, was excluded from the calculations. Peak intensities and partial assignments such as grouping chemical shifts into spin systems or according to atom types (N, Cα, Cβ, Cγ, etc.) within one residue were not used as input for FLYA. For the calculations including the peak lists of the reverse (U-^13^C,^15^N-DGK-I,L,V) labelled sample, the residue types Ile, Leu and Val were excluded. Assignments for ^13^C and ^15^N are counted as correct if they match the manually observed reference assignment within a chemical shift tolerance of 0.55 ppm. The tolerance for chemical shift matching was set to 0.55 ppm as recommended by Schmidt *et al*.^38^. In the ssFLYA results, assignments are classified as strong (reliable; dark colors in Fig. 3b), if ≥80% of the individual chemical shift values from 20 independent runs of the algorithm differ by less than 0.55 ppm from the consensus value (Schmidt *et al*.^38^) Other assignments by ssFLYA are graded as weak (tentative; light colors in Fig. 3b). The assignment calculation could be performed within approximately 10 min using 20 CPU cores in parallel.

#### Analysis of chemical shift perturbations (CSPs) and peak intensities during substrate(s) binding

Scalar and dipolar coupling based experiments of DGK in its apo state, saturated with AMP-PCP, DOG and with AMP-PCP + DOG were carried out for the analysation of CSPs and peak intensities during substrate(s) binding. Concerning the analysis of CSPs in 3D NCACX spectra, weighted chemical shift changes were calculated according to^71^:

$${\rm{\Delta }}\delta ({Cx},\,Ca,\,N)=\sqrt{{({\rm{\Delta }}\delta {Cx})}^{2}+{({\rm{\Delta }}\delta {Ca})}^{2}+{(\frac{{\rm{\Delta }}\delta {\rm{N}}}{2.48})}^{2}},$$

where ΔδCx and ΔδCα and ΔδN represent chemical shift perturbations in the ^13^Cx, ^13^Cα and ^15^N dimension, respectively. All CSPs ≥ 0.2 ppm are counted as significant.

## Supplementary information

Supporting Information

## Data Availability

Data sets generated during and/or analysed during the current study are available from the corresponding author on reasonable request. The chemical shift assignment has been deposited in the BMRB database (access code 27570).
