# Supplementary material for: Global response of diacylglycerol kinase towards substrate binding observed by 2D and 3D MAS NMR
Source: Sci Rep. 2019 Mar 8;9:3995. doi: 10.1038/s41598-019-40264-8 (PMC6408475; doi:10.1038/s41598-019-40264-8)
Supplement: Supplementary file 1 — Supporting Information [file 41598_2019_40264_MOESM1_ESM.pdf]

SUPPORTING INFORMATION

Global response of diacylglycerol kinase towards substrate binding observed by 2D and 3D MAS NMR

Kristin Möbius[1], Sina Kazemi[1], Peter Güntert[1,2,3], Andreas Jakob[4], Alexander Heckel[4], Johanna Becker-Baldus[1] and Clemens Glaubitz[1*]

(1) Institute for Biophysical Chemistry & Centre for Biomolecular Magnetic Resonance, Goethe-University Frankfurt, Germany

(2) Laboratory of Physical Chemistry, ETH Zürich, Switzerland

(3) Graduate School of Science, Tokyo Metropolitan University, Japan

(4) Institute for Chemical Biology and Organic Chemistry, Goethe-University Frankfurt

(*) Correspondence: glaubitz@em.uni-frankfurt.de

Institute for Biophysical Chemistry & Centre for Biomolecular Magnetic Resonance,

Goethe-University Frankfurt

Max-von-Laue-Str. 9

60438 Frankfurt am Main

Germany

Tel.: +49-69-798-29927

Fax.: +49-69-798-29929

www.glaubitz-lab.de

(A) Purity, functionality and structural homogeneity of ssNMR samples

The purity of DGK in DDM micelles was checked by absorption spectroscopy and SDS-PAGE (Fig. S1a), while BN-PAGE analysis offers a reliable assessment of the oligomeric state. It shows DGK exclusively in its trimeric form without any aggregates visible (Fig. S1b). Additionally, a sucrose gradient was carried out. It indicates a homogenous protein reconstitution into the DMPC/DMPA liposomes (Fig. S1c). To verify the functionality of DGK in liposomes, we used a coupled enzyme assay as described before [1,2]. The activities are comparable to those reported previously [2]. They were highly reproducible from sample to sample (n=8, see Tab. S3: wtDGK in DMPC/DMPA in its apo state). To further investigate the quality of the proteoliposome sample, we carried out 1D ^{13}C and ^{15}N cross-polarization (CP) MAS as well as 2D ^{13}C–^{13}C proton driven spin diffusion (PDSD) spectra [3] (Fig. S2b). Despite spectral overlap, which is characteristic for α-helical proteins, a fine structure is clearly observable. The ^{15}N-CP spectrum, for instance, shows a sharp band at approximately 107.0 ppm corresponding to ^{15}N glycine signals (Fig. S2a). The 2D ^{13}C-^{13}C PDSD spectrum was performed with a short carbon-carbon mixing time (20 ms) to provide one-bond correlations between aliphatic atoms (Fig. S2b). It serves as a fingerprint of the sample, evaluating structural homogeneity, resolution and secondary structure. The high spectral resolution demonstrates a homogenous sample preparation. Fig. S2c illustrates the superposition of the PDSD spectrum with the resonance assignment of the thermostable DGK mutant, published by Yang and co-workers [4]. Though this assignment was obtained using MAS NMR as well, significant deviations made a *de-novo* assignment of wild type DGK necessary

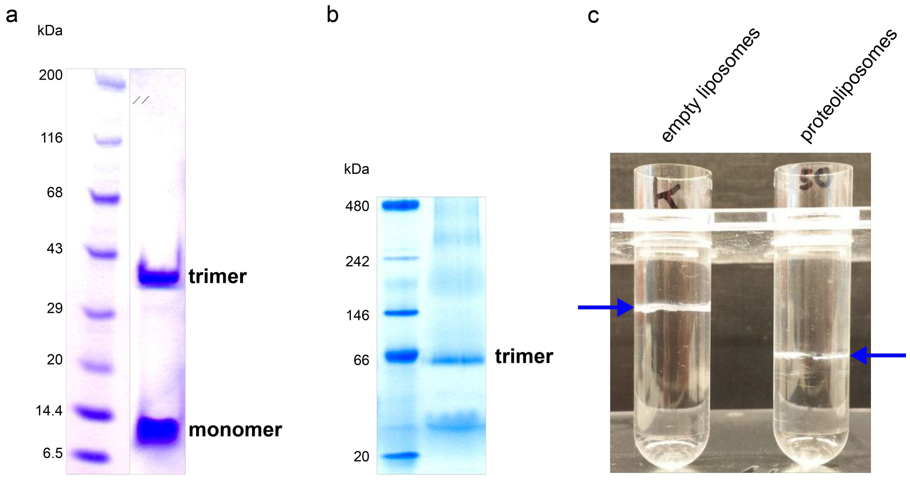

Figure S1: Preparation of *E. coli* diacylglycerol kinase. **(a)** SDS-PAGE of DGK in DDM micelles, verifying the purity of the protein solution after the purification step. Lanes that were non-adjacent in the respective gels are separated by a black line. Full-length gels are presented in Supplementary Figure S9. **(b)** BN-PAGE of DGK in DDM micelles, clearly showing DGK in its trimeric form. **(c)** Sucrose density gradient (40-10 %) of DGK reconstituted in DMPC/DMPA, illustrating a homogeneous size distribution of proteoliposomes without any empty liposomes observable.

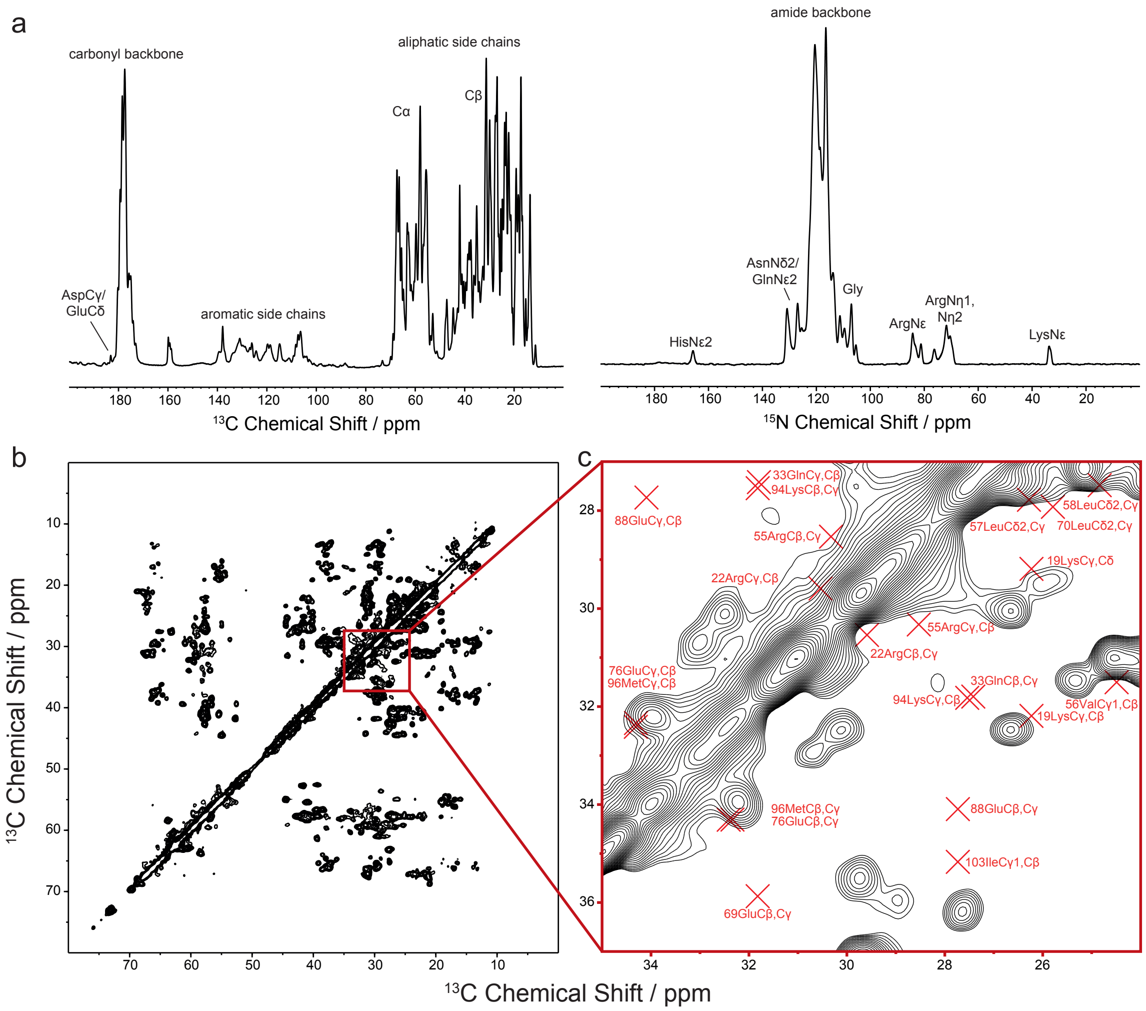

Figure S2: MAS NMR spectra of DGK embedded within lipid bilayers recorded at a MAS rate of 15.2 kHz and a temperature of 275 K. **(a)** 1D ^{13}C and ^{15}N cross polarization (CP) spectra of U-^{13}C,^{15}N-wtDGK, showing a fine structure with single well-resolved resonances. A contact time of 1.25 ms was used.**(b)** 2D ^{13}C-^{13}C PDSD spectrum of U-^{13}C,^{15}N-wtDGK. A mixing time of 20 ms was used. The high number of well-resolved peaks indicates a homogeneous sample preparation. **(c)** Enlargement of the selected region in the 2D ^{13}C-^{13}C PDSD spectrum. The spectrum is compared with the assignment of the thermostable mutant of DGK obtained by MAS ssNMR [4]. The comparison reveals that a transfer of these assignments to the wild-type sample is not possible. The red cross peaks were generated with CCPN analysis 2.4.1 [5].

(B) Comparison of different liposome compositions

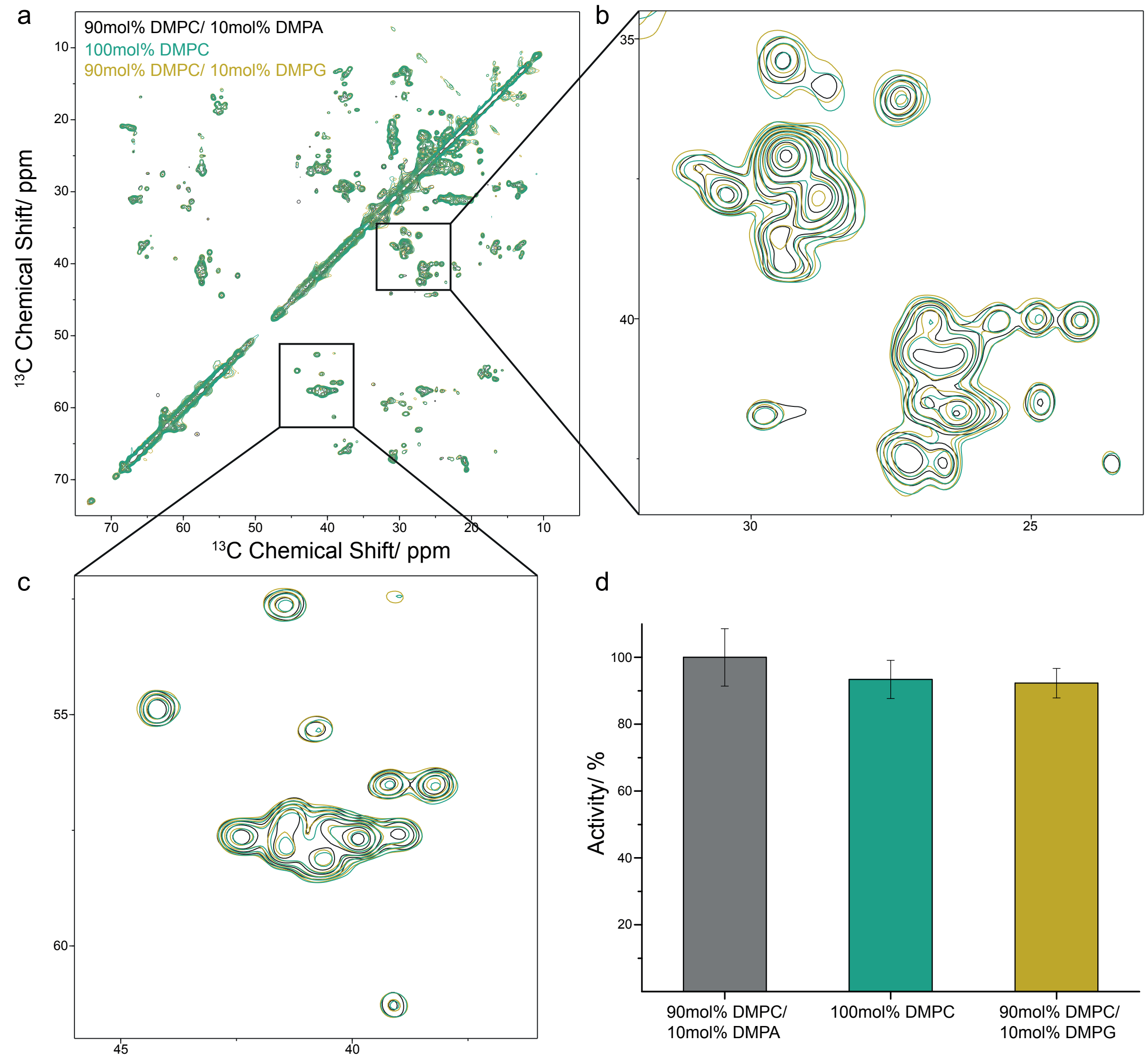

Figure S3: Comparison of DGK embedded in different liposome compositions. **(a)** Superposition of 2D ^{13}C-^{13}C PDSD spectra of U-^{13}C,^{15}N-wtDGK reconstituted into 90mol% DMPC/ 10mol% DMPA (black), 100mol% DMPC (green) and 90mol% DMPC/ 10mol% DMPG (yellow). The NMR spectra were recorded at 275 K. **(b and c)** Enlargement of the respective selected regions in the 2D ^{13}C-^{13}C PDSD spectra. The comparison reveals a similar fingerprint for DGK in all three liposome compositions. **(d)** Activity of DGK reconstituted in 90mol% DMPC/ 10mol% DMPA (black), 100mol% DMPC (green) and 90mol% DMPC/ 10mol% DMPG (yellow) demonstrating a similar activity in all three liposome compositions. The activity data were acquired at 30 °C.100% activity corresponds to the rate recorded with wtDGK in 90mol% DMPC/ 10mol% DMPA of 90 (± 9.9) µmol min^{-1} mg^{-1}. Experiments were repeated three times. The activity was calculated as the mean value. Error bars correspond to standard deviations.

(C) Supplementary MAS NMR data

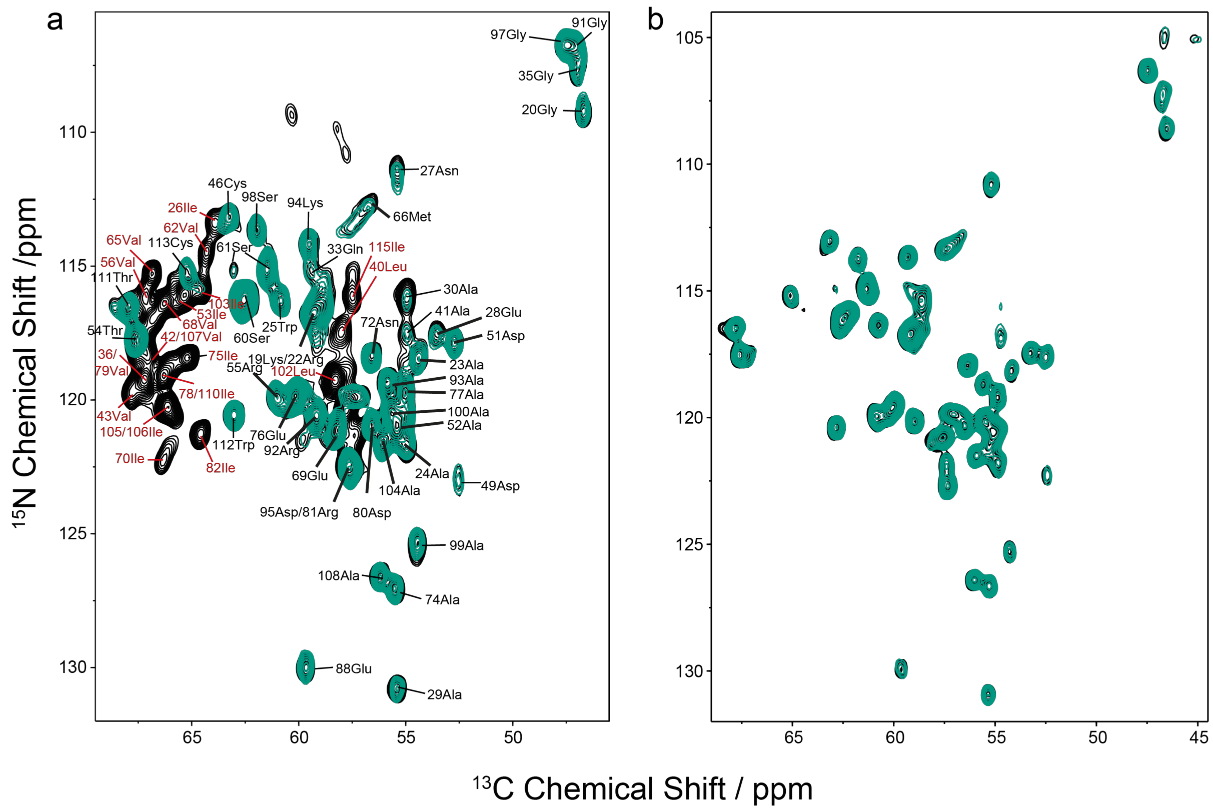

Figure S4: 2D NCA spectra of DGK. **(a)** Comparison of 2D NCA spectra of uniform labelled U-^{13}C,^{15}N-DGK (black) and reverse labelled U-^{13}C,^{15}N-DGK-I,L,V (green). The ^{15}N and ^{13}Cα signals of the uniform labelled sample show narrow lines with approximately 105 and 185 Hz linewidths, respectively. To resolve residual ambiguities, Ile, Leu and Val are specifically unlabelled. Ile, Leu and Val are highlighted in red. They disappear as expected in the spectrum of the reverse labelled sample. **(b)** 2D NCA spectra of U-^{13}C,^{15}N-DGK-I,L,V incubated with 14 mM Mg*AMP-PCP (pH 7.2) recorded immediately after the incubation (black) and after 30 d (green) demonstrate that the fully saturated system is stable over a long period of time without any significant signs of degradation.

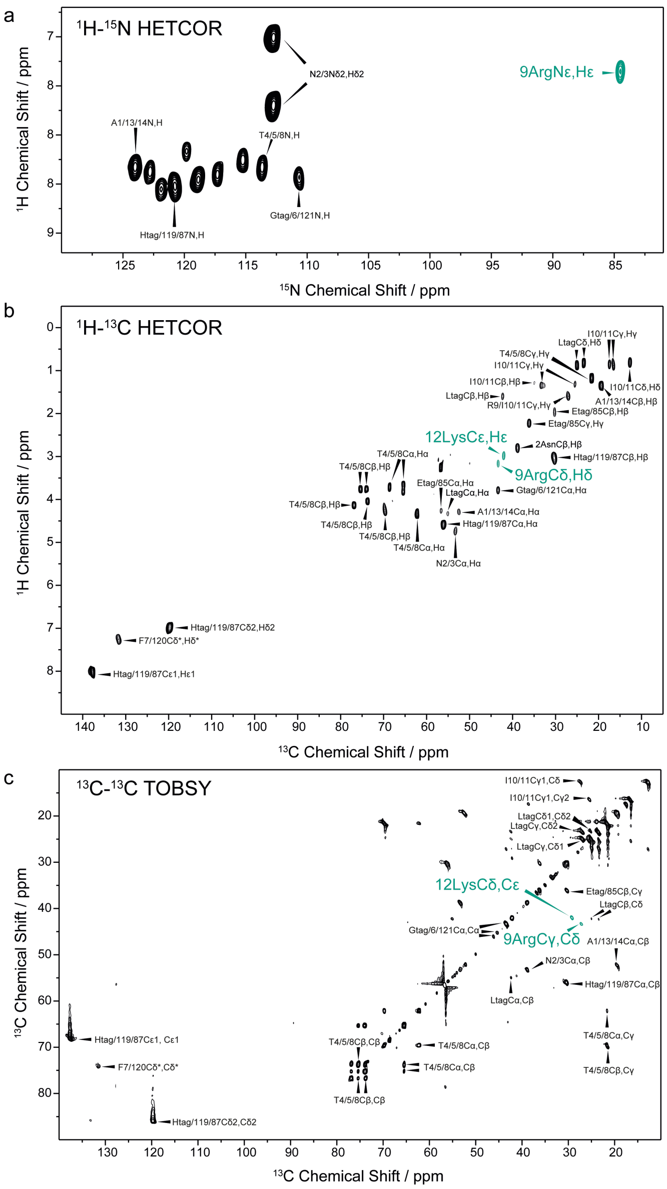

Figure S5: 2D scalar-coupling based ^{1}H-^{15}N HETCOR **(a)**, ^{1}H-^{13}C HETCOR **(b)** and ^{13}C-^{13}C TOBSY **(c)** of U-^{13}C,^{15}N-DGK with residue-specific assignments. All residues, which could not be detected and assigned by dipolar-coupling based experiments are considered as possible candidates for detection by J-based experiments. INEPT and TOBSY were used for ^{1}H-^{15}N or ^{1}H-^{13}C heteronuclear polarization and ^{13}C-^{13}C homonuclear mixing, respectively. Peaks for Arg9 and Lys12 are highlighted green, since they could be assigned unambiguously. Peaks for the aromatic rings were folded in the indirect dimension to save experimental time. Amino acids that correspond to the His-tag are labelled by 'tag'.

(D) Secondary structure analysis

Based on the chemical shift assignment, a secondary structure analysis was carried out using the chemical shift index [6]. It is compared to the DGK X-ray structure (PDB 3ZE4, chain A) [7] and to the secondary structure obtained by MAS NMR for a thermostable DGK-mutant [4] in Fig. S6. All three structures feature substantial similarities, especially concerning the high α-helical content. However, there are few differences. The crystal structure shows small deviations around the interhelical turn (T) between helix 1 (H1) and the surface helix (SH), around the periplasmic loop (PL) between helix 1 (H1) and helix 2 (H2), as well as the cytoplasmic loop (CL) between helix 2 (H2) and helix 3 (H3). In subunit A of the crystal structure, the position of T and PL is slightly shifted upstream by two residues compared to the MAS NMR structures. Additionally, T is one residue longer and PL one residue shorter in the X-ray structure than in the MAS NMR structures. Concerning the position and/or length of the CL, all three structures vary from each other. CL is shifted from the residues 83-87 in the MAS NMR structure of wtDGK to the residues 81–85 in the MAS NMR structure of the thermostable mutant and to the residues 83–90 (subunit A) of the X-ray structure of wtDGK. However, it has to be noted that the positions and lengths of the non-helical structures are even inconsistent between the three different subunits A, B and C within the crystal structure [4,7].

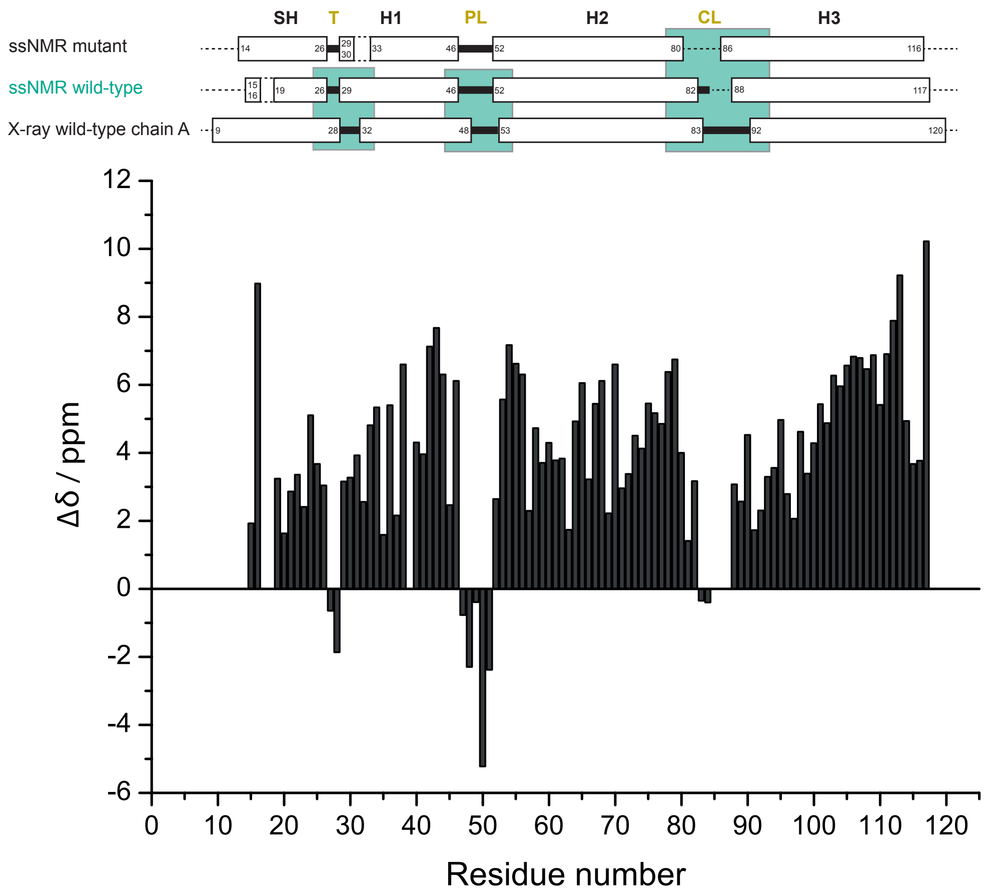

Figure S6: Chemical shift based secondary structure analysis. The chemical shift index (CSI) $\Delta\delta$ is derived from the deviation of the experimentally determined MAS NMR chemical shifts (exp) for Cα and Cβ from their random coil standard chemical shifts (rc) according to $\Delta\delta = [\delta C\alpha(exp)-\delta C\alpha(rc)] - [\delta C\beta(exp)-\delta C\beta(rc)]$ [6]. For Gly residues and residues without any assignment of Cβ, only Cα secondary shifts were considered. Strongly positive (≥ 1.5 ppm) values of the CSI indicate an α-helical structure, whereas negative or near-zero values imply deviations from helicity. In addition, the secondary structure of wild-type DGK determined by ssNMR is compared with the ssNMR structure of the thermostable mutant [4] and the crystal structure of wtDGK (PDB 3ZE4, chain A) [8]. Rectangles symbolize α-helical regions including the surface helix (SH) and the three transmembrane helices (H1-3), whereas solid lines reflect deviations from helicity including the interhelical turn (T), the periplasmic (PL) as well as the cytoplasmic loop (CL). Residues that were not resolved by ssNMR or by X-ray crystallography are illustrated by dashed lines. Differences between the secondary structures obtained by X-ray crystallography and ssNMR are highlighted in green.

(E) DOG bound state of DGK

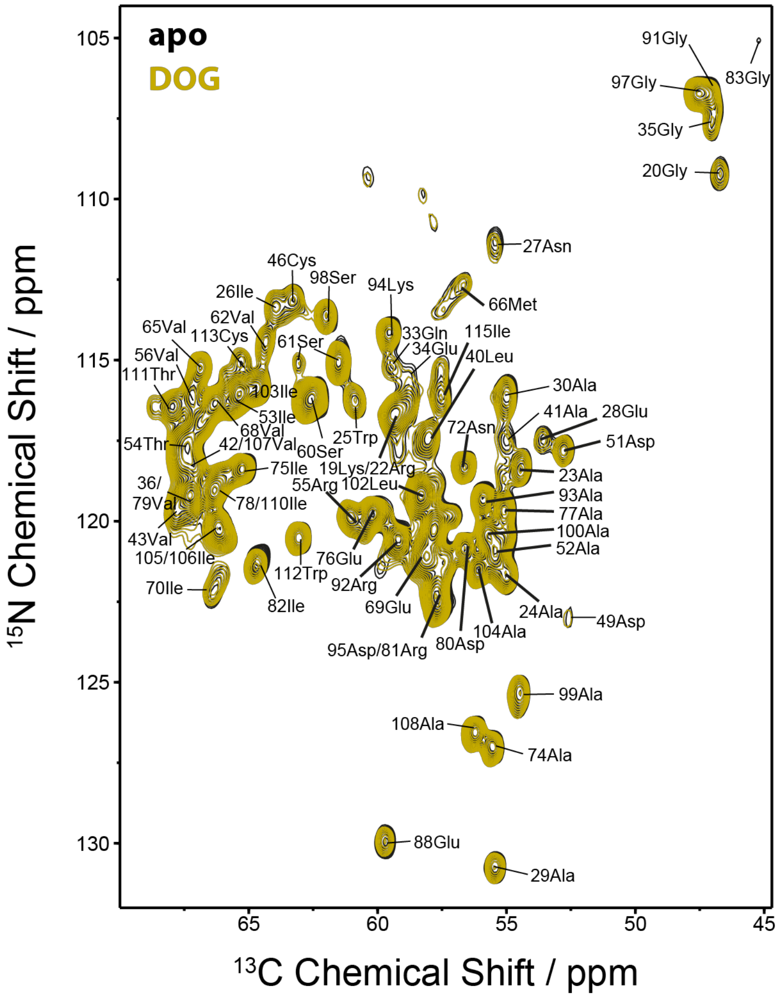

Figure S7: Effect of DOG binding on DGK. Superposition of 2D NCA spectra of DGK's apo (black) and DOG-bound (yellow) state. Both spectra overlap demonstrating that the presence of DOG causes only minor chemical shift changes.

(F) Supplementary data on inter-protomer contacts

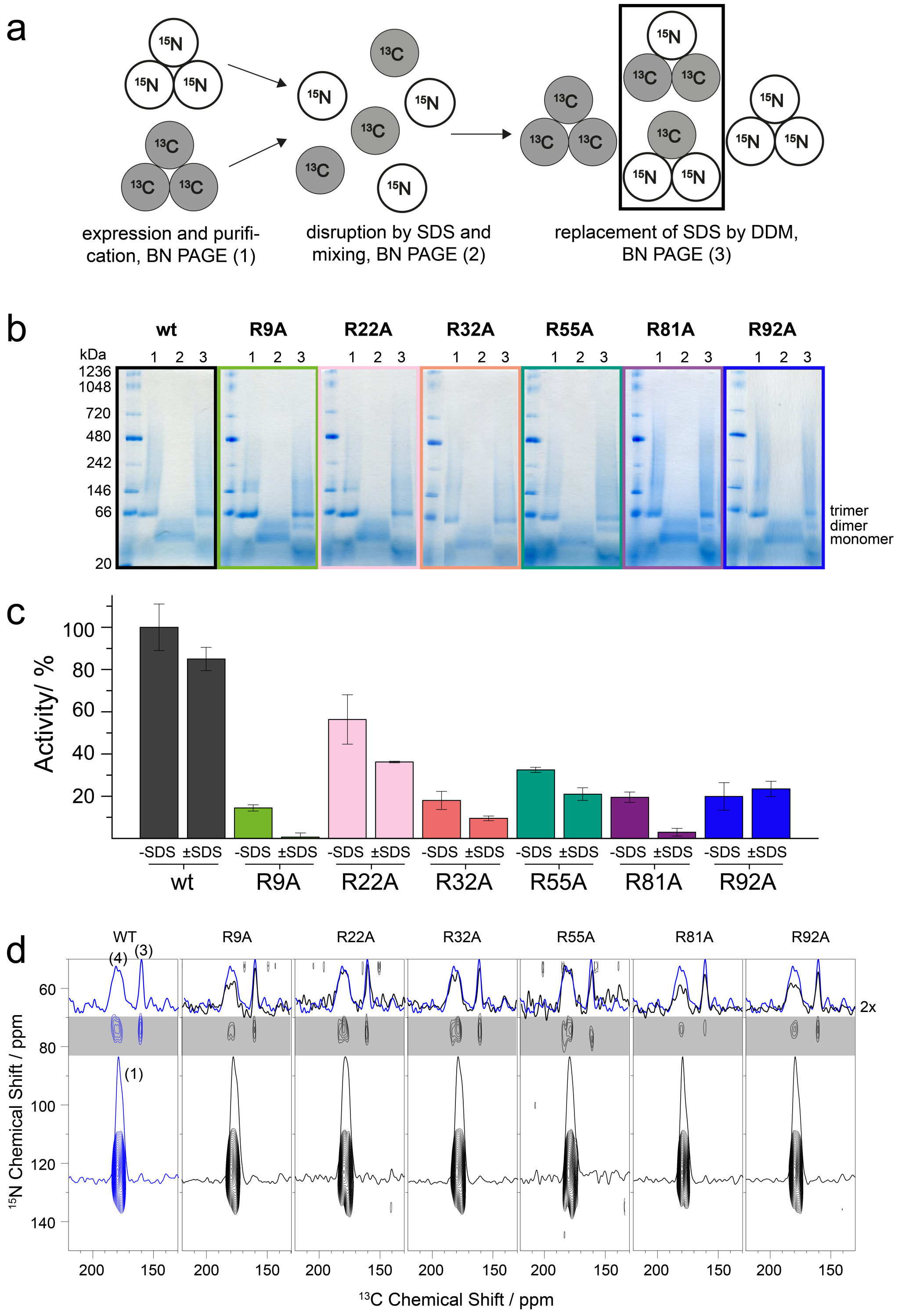

Figure S8: Preparation and analysis of mixed-labelled DGK trimers and the effect of single Arg mutations. (a) Differently labelled trimers of DGK are separately expressed, solubilized, purified and eluted in DDM. Then, they are disrupted into monomers or dimers by SDS and mixed in a 1:1 ratio. Afterwards, SDS is replaced by DDM, leading to mixed labelled DGK trimers, which can then be reconstituted into a lipid bilayer. Assembling [CN]-DGK from ^{13}C (gray) and ^{15}N (white) labelled monomers leads to 4 distinct trimer configurations, which are differently populated. The average number of NC interfaces per trimer is 1.5, of which only 50 % are unique (N → C *vs.* C → N). Due to this statistical distribution, the number of potential cross-protomer interactions involving residues with ^{13}C-labelled side chains on the one side and ^{15}N-labelled side chains on the other is small, making the use of DNP essential. (b) BN-PAGE analysis on DGK and its RxA mutants verifies successful trimer

formation after disruption and mixing as shown in (a). Trimers are seen in DDM micelles (1), which are disrupted by SDS (2) but form again after detergent exchange to DDM (3). All RxA mutants feature a similar oligomerization behavior as the wild-type, suggesting that the respective arginines, which are all located in extramembranous regions of DGK, are not essential for the trimer formation. For each DGK sample a new gel was prepared and processed the same way. Lanes that were non-adjacent in the respective gels are separated by a black line. Full-length gels are presented in Supplementary Figure S9. (c) Coupled activity assay with wtDGK and RxA-DGK embedded into lipids, showing a reduction of activity for all Arg-mutants compared to the wild-type. This in turn displays the importance of all arginines for the catalytic activity. DGK trimers from DDM micelles (-SDS) and DGK trimers from DDM micelles after SDS treatment (±SDS) were reconstituted into lipid bilayers and then measured. Almost full activity (85%) is regained after detergent exchange and subsequent trimer formation, which is in agreement with previously reported activity data based on unfolding/refolding experiments on wtDGK [9]. The activity was measured three times and calculated as the mean value. Error bars correspond to standard deviations. (d) DNP-enhanced ^{15}N−^{13}C-TEDOR spectra of mixed labelled trimers (U-^{13}C/U^{15}N^{12}C)-DGK and its Arg-mutants. A mixing time of 6.25 ms (24 rotor cycles) was used for all experiments, which is optimal for N-C distances between 2.5-3 Å. Three crosspeaks can be identified: Intra-protomer natural abundance N-CO contact (1), intra-residue natural abundance contact between ArgNη,ε -Cζ (3) and cross-protomer contacts between ArgNη,ε and GluCδ/AspCγ/AsnCγ (4). All spectra are normalized with respect to the N-CO crosspeak. A reduction in the intensity of resonance (2) is observed upon introducing the R9A, R81A and R92A mutation, while no significant effect is seen for R22A, R32A and R55A. These data show, that all three residues R9, R81 and R92 are involved in cross-protomer interactions. These findings are in principle compatible with the 3D crystal structure [8], in which these residues have suitable locations at the interface to be involved in such interactions. Slices through the crosspeaks were created by integrating along the ^{15}N dimension from 68 to 84 ppm and 108 to 138 ppm, respectively

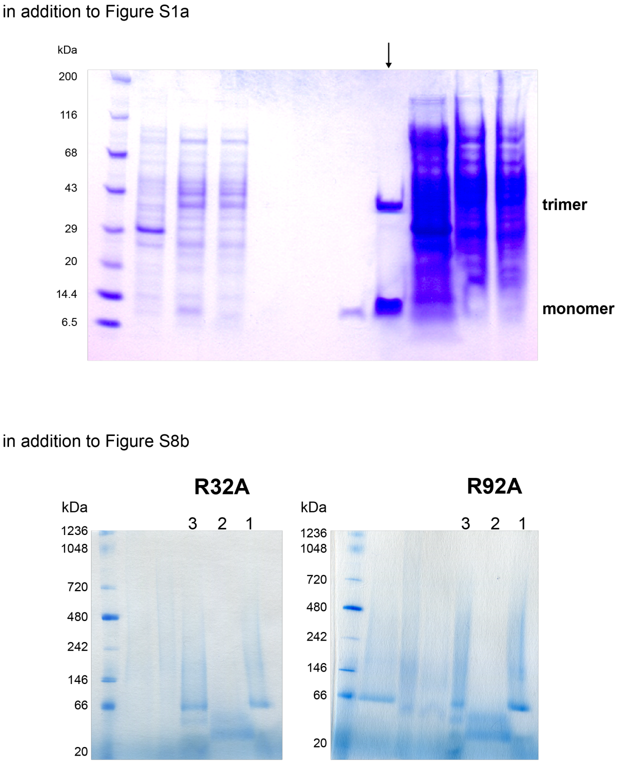

Figure S9: Full display of SDS PAGE in addition to Suppl. Figure S1a (top) and full display of BN-PAGE in addition to Suppl. Figure S8b (bottom).

(E) Supplementary tables

Table S1: Resonance assignments of wild-type DGK in DMPC/DMPA liposomes by MAS NMR experiments. Chemical shifts are given in ppm.

ResID	N	C‘	Cα	Cβ	Cδ	Cδ1	Cδ2	Hδ	Cε	Cε2	Cε3	Nε	Hε	Cγ	Cγ1	Cγ2	Ch2	Nη1/2	Cζ	Cζ2	Cζ3	Nζ
1 Ala																						
2 Asn																						
3 Asn																						
4 Thr																						
5 Thr																						
6 Gly																						
7 Phe																						
8 Thr																						
9 Arg					43.30			3.16				84.50	7.35	27.24				71.69				
10 Ile																						
11 Ile																						
12 Lys					29.21				42.05				2.98									
13 Ala																						
14 Ala		174.70																				
15 Gly	113.27	177.97	47.07																			
16 Tyr	119.52	176.61	59.46	31.06		133.85				117.47												
17 Ser																						
18 Trp																						
19 Lys	116.77	180.05	59.25	32.57										25.75								
20 Gly	109.11	174.01	46.56																			
21 Leu	121.55	177.73	57.50	41.67		23.45								26.02								
22 Arg	120.40	177.03	59.16	29.78	44.30									29.82								
23 Ala	118.36	180.19	54.41	18.39																		
24 Ala	121.80	176.86	54.81	15.75																		
25 Trp	116.31	176.51	60.75	29.28		126.59								110.67								
26 Ile	113.33	177.81	63.94	38.49		13.28									28.84	17.01						
27 Asn	111.21	174.96	55.36	41.22										176.90								
28 Glu	117.46	174.69	53.42	28.63	180.87									32.91								
29 Ala	130.72	178.95	55.33	18.24																		
30 Ala	116.25	179.25	54.83	18.01																		

31 Phe	114.63	176.77	61.36	39.31		131.20								139.33								
32 Arg	116.47	178.04	58.93	31.50	43.94							83.32		27.90				69.98	158.75			
33 Gln	115.09	179.52	59.36	27.86										33.84								
34 Glu	115.68	177.02	59.05	26.95										34.39								
35 Gly	107.72	174.59	46.99																			
36 Val	119.17	176.50	67.07	32.15											21.55	21.55						
37 Ala	118.85	178.14	54.92	19.35																		
38 Val	117.04	178.16	67.30	31.08																		
39 Leu	117.44	178.32																				
40 Leu	117.35	177.74	58.25	41.14										26.94								
41 Ala	117.24	179.25	55.11	18.01																		
42 Val	118.13	177.96	67.26	31.02											22.94	22.94						
43 Val	119.61	177.79	67.72	30.75											22.26	22.26						
44 Ile	118.74	177.77	65.96	37.56		13.33									29.75	17.01						
45 Ala	120.51	179.15	54.95	18.83																		
46 Cys	113.21	172.57	63.16	27.62																		
47 Trp	121.36	176.97	58.17	30.72																		
48 Leu	116.03	176.56	54.88	44.48		23.35								26.37								
49 Asp	123.00	176.03	52.59	39.28																		
50 Val	109.85	174.87	58.02	33.38											20.29	17.35						
51 Asp	117.78	174.53	52.71	41.67										180.04								
52 Ala	121.13	178.54	55.39	19.18																		
53 Ile	116.01	177.28	65.31	37.14		13.35									29.67	18.89						
54 Thr	115.95	175.45	67.45													21.12						
55 Arg	119.97	177.62	60.87	29.37	42.70									27.97								
56 Val	116.13	178.36	67.23	31.01											24.37	23.39						
57 Leu	122.24	179.87	57.67	42.60		26.62																
58 Leu	120.34	179.05	57.67	39.96		25.74	24.39							27.02								
59 Ile	116.14	178.89	65.29	38.99		16.12									29.00	17.93						
60 Ser	116.17	177.13	62.56																			
61 Ser	115.07	177.34	61.49	63.01																		
62 Val	114.40	177.57	64.24	30.41											23.31	19.38						
63 Met	119.86	179.25	57.15	32.89										30.61								
64 Leu	120.25	178.18	57.71	39.98		22.43								25.13								

65 Val	115.30	176.57	66.77	31.18											21.99	20.26						
66 Met	112.89	177.98	56.64	31.47																		
67 Ile	119.09	176.63	65.77	38.01		14.24									28.93	17.25						
68 Val	116.36	177.23	66.70	31.02											23.23	22.87						
69 Glu	120.91	179.11	58.01	29.52	181.23									35.33								
70 Ile	122.38	177.26	66.32	37.18		14.83									26.56	19.48						
71 Leu	121.20	178.28	57.95	41.68		22.05								26.56								
72 Asn	118.20	176.83	56.50	38.45										178.26								
73 Ser	115.97	176.06	62.25	63.04																		
74 Ala	126.91	178.33	55.37	17.54																		
75 Ile	118.39	177.28	65.09	36.97		12.81									29.34	16.61						
76 Glu	119.75	176.88	59.99	28.77	182.67									35.65								
77 Ala	119.70	178.92	55.01	17.00																		
78 Val	116.34	177.02	66.27	30.86											23.53	22.96						
79 Val	119.21	179.05	67.17	30.95											24.33	22.95						
80 Asp	120.95	178.21	56.46	39.41																		
81 Arg	122.54	178.18	57.45		42.24							84.30		27.50				71.70	158.13			
82 Ile	121.11	176.84	64.49	37.96		15.16									28.86	16.32						
83 Gly	105.00	172.69	44.96																			
84 Ser	121.37	179.12	58.34																			
85 Glu																						
86 Tyr																						
87 His																						
88 Glu	129.84	179.21	59.55	29.52										35.36								
89 Leu	121.29	179.02	57.31	41.66										27.08								
90 Ser	116.49	175.47	62.30	62.56																		
91 Gly	106.74	174.75	47.04																			
92 Arg	120.47	177.89	59.02	31.36	44.27							80.77		25.16				76,04/69,99	159.39			
93 Ala	119.23	179.12	55.82	18.80																		
94 Lys	114.08	179.99	59.41	32.43	29.86				41.71					26.55								
95 Asp	122.43	178.72	57.58	39.11																		
96 Met	119.97	177.09	60.26	33.92					16.91					32.09								
97 Gly	106.66	175.57	47.55																			
98 Ser	113.65	177.28	61.82	62.57																		

99 Ala	125.37	178.14	54.34	17.85																		
100 Ala	120.41	178.62	55.58	17.94																		
101 Val	116.25	177.43	66.17	31.30											21.17							
102 Leu	119.23	178.20	58.16	40.74		23.80	22.02							27.04								
103 Ile	115.82	177.00	64.63	36.09		11.07									27.45	18.79						
104 Ala	121.52	179.59	55.96	17.02																		
105 Ile	120.16	149.30	66.03	37.44		12.98									30.73	16.95						
106 Ile	120.13	177.49	66.12	37.14		12.92									29.40	16.71						
107 Val	118.35	178.58	67.08	31.06											22.88	22.88						
108 Ala	126.54	178.22	56.07	16.67																		
109 Val	117.62	178.17	67.19	30.99											22.00	22.91						
110 Ile	118.96	176.72	66.29	38.40		13.27									29.78	17.04						
111 Thr	116.61	175.14	67.90	68.50												20.77						
112 Trp	120.50	177.88	62.90	27.39		125.68	130.41			137.55	118.98	130.66					123.76			114.76	120.84	
113 Cys	115.39	176.33	64.95	27.45																		
114 Ile	116.27	178.76	65.74	37.70		13.75									27.38							
115 Leu	116.25	180.15	57.44	40.71		22.52								26.63								
116 Leu	118.76	178.24	57.69	40.39		21.84								27.09								
117 Trp	115.61	175.24	64.57	26.34																		
118 Ser	115.44	175.38	65.90																			
119 His																						
120 Phe																						
121 Gly																						

Table S2: Summary of all significant perturbations in peak position and intensity during the interaction of DGK with AMP-PCP. An increase/decrease of at least 20% was considered as significant.

			AMP-PCP	
			weighted CSP	peak intensity
15	Gly	Cα	x	disappeared
		C’	x	disappeared
16	Tyr	Cα	x	disappeared
		Cβ	x	disappeared
		Cδ1	x	disappeared
		Cε2	x	disappeared
		C‘	x	disappeared
19	Lys	Cβ	x	disappeared
		Cγ		
		C‘	x	reduced
20	Gly	Cα	0.22	increased
		C‘	0.22	increased
22	Arg	Cδ	x	disappeared
23	Ala	Cβ	0.35	x
		C‘	0.26	x
25	Trp	Cγ	0.38	x
26	Ile	Cβ	0.22	reduced
		Cγ1	0.22	x
		Cγ2	x	increased
		Cδ	x	increased
27	Asn	Cβ	0.20	x
		Cγ	0.21	x
28	Glu	Cγ	0.40	reduced
		Cδ		
		C‘	0.21	x
29	Ala	Cα	0.22	x
		Cβ	0.23	x
		C‘	0.31	x
31	Phe	Cβ	0.27	x

		**Cγ**	0.58	reduced
		**Cδ**	0.57	x
		**Cε**	x	appears
**32**	**Arg**	**Cβ**	0.24	reduced
		**Cγ**	0.41	x
		**Cδ**	0.25	reduced
**33**	**Gln**	**Cβ**	x	reduced
		**Cγ**	0.54	reduced
		**C'**	0.23	reduced
**37**	**Ala**	**Cα**	x	reduced
		**Cβ**	0.21	reduced
		**C'**	x	reduced
**40**	**Leu**	**Cδ**	x	appears
**41**	**Ala**	**Cβ**	0.30	x
**43**	**Val**	**Cβ**	0.35	x
		**Cγb**	0.36	x
**45**	**Ala**	**Cβ**	0.22	x
		**C'**	0.47	x
**46**	**Cys**	**Cα**	0.23	reduced
		**Cβ**	0.27	reduced
		**C'**	0.22	reduced
**47**	**Trp**	**Cβ**	x	disappeared
**48**	**Leu**	**Cδ**	0.24	x
**49**	**Asp**	**Cα**	0.34	x
		**Cβ**	0.38	increased
		**C'**	1.29	increased
**50**	**Val**	**Cα**	x	disappeared
		**Cβ**	x	disappeared
		**Cγb**	x	disappeared
		**Cγa**	x	disappeared
		**C'**	x	disappeared
**51**	**Asp**	**Cα**	x	reduced
		**Cβ**	x	reduced

		Cγ	0.33	reduced
		C‘	x	reduced
53	Ile	Cγ2	0.40	x
		C‘	0.28	x
55	Arg	Cβ	0.27	reduced
		Cγ	x	reduced
		Cδ	0.28	reduced
56	Val	Cγ1	0.32	x
57	Leu	C‘	0.22	reduced
62	Val	Cβ	0.38	x
		Cγ2	0.23	x
		Cγ1	0.43	x
65	Val	C‘	0.29	x
66	Met	Cα	0.21	reduced
		Cβ	x	reduced
		C‘	0.27	x
67	Ile	Cγ1	x	reduced
		Cδ	x	reduced
69	Glu	Cβ	1.20	reduced
		Cγ	x	disappeared
		Cδ	x	disappeared
70	Ile	Cα	0.28	x
		Cβ	0.38	x
		Cγ1	0.31	x
		Cγ2	0.39	x
		Cδ	0.47	increased
		C‘	0.27	x
72	Asn	Cα	0.20	x
74	Ala	Cβ	0.32	x
76	Glu	Cβ	x	reduced
		Cγ	0.26	reduced
		Cδ	x	disappeared

**77**	**Ala**	**C‘**	0.22	x
**79**	**Val**	**Cβ**	0.28	x
		**Cγ1**	0.45	x
		**Cγ2**	0.28	x
		**C‘**	0.44	x
**80**	**Asp**	**Cα**	0.36	x
		**Cβ**	0.25	x
		**C‘**	0.28	x
**81**	**Arg**	**Cγ**	0.39	x
		**Cδ**	0.62	x
		**Cζ**	x	reduced
**82**	**Ile**	**Cα**	0.62	reduced
		**Cβ**	0.44	reduced
		**Cγ1**	0.43	reduced
		**Cγ2**	0.54	reduced
		**Cδ2**	0.48	reduced
		**C‘**	0.54	reduced
**83**	**Gly**	**Cα**	x	increased
		**C‘**	x	increased
**88**	**Glu**	**Cα**	0.39	reduced
		**Cβ**	0.29	reduced
		**Cγ**	x	disappeared
		**C‘**	0.21	reduced
**91**	**Gly**	**Cα**	0.70	x
		**C‘**	0.80	x
**92**	**Arg**	**Cα**	x	reduced
		**Cβ**	0.24	reduced
		**Cγ**	x	reduced
		**Cδ**	x	reduced
		**C‘**	x	reduced
**94**	**Lys**	**Cβ**	0.27	x
		**Cγ**	0.28	x
		**Cδ**	0.76	x
		**Cε**	0.20	x
**95**	**Asp**	**Cβ**	x	reduced

**97**	**Gly**	**Cα**	0.23	x
		**C'**	0.31	x
**98**	**Ser**	**Cα**	x	reduced
		**Cβ**	x	reduced
		**C'**	x	reduced
**99**	**Ala**	**Cα**	x	reduced
		**Cβ**	x	reduced
		**C'**	x	reduced
**101**	**Val**	**Cγ1**	0.30	x
**102**	**Leu**	**Cα**	0.29	x
		**Cδ1**	0.21	x
**111**	**Thr**	**C'**	0.28	x
**114**	**Ile**	**Cγ1**	x	increased
		**Cδ**	x	increased
**116**	**Leu**	**Cα**	x	disappeared
		**Cβ**	x	reduced
		**Cγ**	x	reduced
		**Cδ**	x	disappeared
		**C'**	x	disappeared

Table S3. Catalytic activity of different DGK samples used in this study. For each sample, the activity was measured three times and calculated as the mean value.

sample		activity in DMPC (90mol%)/ DMPA (10mol%) [µmol * min^{-1} * mg^{-1}]
labelled DGK for measurements at high field	U-^{13}C,^{15}N-wtDGK	102.8 ± 12.4
		86.2 ± 5.4
		90.0 ± 11.5
		77.9 ± 13.6
	U-^{13}C,^{15}N-wtDGK/I,L,V	78.3 ± 14.4
		99.6 ± 10.1
unlabelled DGK	wtDGK + 0 mM AMP-PCP	89.2 ± 9.7
	wtDGK + 4 mM AMP-PCP	58.1 ± 1.3
	wtDGK + 8 mM AMP-PCP	29.0 ± 0.5
	wtDGK + 10 mM AMP-PCP	6.0 ± 0.05
	wtDGK + 12 mM AMP-PCP	9.2 ± 0.1
	wtDGK + 14 mM AMP-PCP	2.9 ± 0.01
	wtDGK + 16 mM AMP-PCP	3.8 ± 0.03
	wtDGK + 0 mM AMUPol	92.4 ± 3.7
	wtDGK + 20 mM AMUPol	89.1 ± 8.9
	DGK-R9A	13.0 ± 0.2
	DGK-R22A	50.7 ± 5.9
	DGK-R32A	16.2 ± 0.7
	DGK-R55A	29.2 ± 0.4
	DGK-R81A	17.6 ± 0.4
	DGK-R92A	17.9 ± 1.2
mixed labelled DGK trimers for DNP-enhanced TEDOR measurements	[CN(Arg)]-DGK	76.5 ± 4.2
	[CC]-DGK	72.0 ± 8.6
	[CN]-DGK	78.3 ± 6.9
	[CN]-DGK-R9A	0.52 ± 0.01
	[CN]-DGK-R22A	27.7 ± 1.0
	[CN]-DGK-R32A	7.3 ± 0.08
	[CN]-DGK-R55A	16.1 ± 0.5
	[CN]-DGK-R81A	2.3 ± 0.04
	[CN]-DGK-R92A	18.0 ± 0.65

<table>
<tr><th colspan="2">sample</th><th>activity in DMPC (100mol%)
[µmol * min^{-1} * mg^{-1}]</th></tr>
<tr><td rowspan="4">labelled DGK for measurements at high field</td><td rowspan="4">U-^{13}C,^{15}N-wtDGK</td><td>84.1 ± 4.8</td></tr>
<tr><th>activity in DMPC (90mol%)/ DMPG (10mol%)
[µmol * min^{-1} * mg^{-1}]</th></tr>
<tr><td>83.1 ± 3.7</td></tr>
</table>

Table S4: Experimental parameters for all multidimensional and dipolar coupling based spectra of DGK in its apo state (white), saturated with AMP-PCP (light grey), DOG (middle grey) and with AMP-PCP + DOG (dark grey).

dimensionality	2D						
experiment	PDSD	DARR	NCA				
figure	Fig. S2b, S2c, S3a	Fig. 6a	Fig. S4a	Fig. 2b, 5a, 5f, S4a, S7	Fig. 5a, 5g, S4b	Fig. S7	Fig. 5f, 5g
sample	U-^{13}C,^{15}N-DGK	U-^{13}C,^{15}N-DGK-**I,L,V**	U-^{13}C,^{15}N-DGK-**I,L,V**	U-^{13}C,^{15}N-DGK	U-^{13}C,^{15}N-DGK + **AMP-PCP**	U-^{13}C,^{15}N-DGK + **DOG**	U-^{13}C,^{15}N-DGK + **AMP-PCP + DOG**
probehead	HCN	E-free	E-free	HCN	E-free	E-free	HCN
recycle delay [s]	2.5	0.8	0.8	3.5	1.0	0.8	2.5
transfer 1	HC-CP	HC-CP	HC-CP	HN-CP	HN-CP	HN-CP	HN-CP
field [kHz]	84(H) 55.6(C)	75.7(H) 55.6(C)	70.9(H) 55.6(C)	72.8(H) 41.7(N)	74.3(H) 41.7(N)	72.1(H) 41.7(N)	76.7(H) 41.7(N)
shape (ramp)	80.100 (H)	80.100 (H)	80.100 (H)	80.100 (H)	80.100 (H)	80.100 (H)	80.100 (H)
contact time [ms]	1.25	1.20	1.30	1.40	1.40	1.50	1.20
carrier [ppm]	113	113	118.2	118.2	118.2	118.2	118.2
transfer 2	PDSD	DARR	NCA-DCP	NCA-DCP	NCA-DCP	NCA-DCP	NCA-DCP
field [kHz]	-	11.8(H)	38(N) 22.8(C) 83.3(H)	38(N) 22.8(C) 100(H)	38(N) 22.8(C) 83.3(H)	38(N) 22.8(C) 83.3(H)	38(N) 22.8(C) 83.3(H)
shape (ramp)			90.100 (C)	90.100 (C)	90.100 (C)	90.100 (C)	90.100 (C)
contact/mixing time [ms]	20	800	4.8	3.5	5.0	4.4	3.8
carrier [ppm]			60.9	60.9	60.9	60.9	60.9
T1 increments	1344	1344	176	160	165	176	160
spectral width [kHz]	55.6	55.6	3.8	3.8	3.8	3.8	3.8
aqu. time [ms]	12.0	12.0	23.2	21.0	21.7	23.2	21.1
T2 increments	3390	3390	3380	3390	3380	3380	3380
spectral width [kHz]	100	100	100	100	100	100	100
aqu. time [ms]	17	17	17	17	17	17	17
^{1}H SPINAL decoupling [kHz]	100	83.3	83.3	100	83.3	83.3	83.3
number of scans	72	192	496	208	336	336	128
total measurement time	2d21h	4d21h	20h	1d9h	16h	14h	14h

dimensionality	2D		
experiment	NCACX		NCOCX
figure	Fig. 5b	Fig. 5b	Fig. 6b
sample	U-^{13}C,^{15}N-DGK	U-^{13}C,^{15}N-DGK + **AMP-PCP**	U-^{13}C,^{15}N-DGK
probehead	HCN	E-free	E-free
recycle delay [s]	3.0	1.0	1.2
transfer 1	HN-CP	HN-CP	HN-CP
field [kHz]	88(H) 41.7(N)	74.3(H) 41.7(N)	73.3(H) 41.7(N)
shape (ramp)	80.100 (H)	80.100 (H)	80.100 (H)
contact time [ms]	1.4	1.4	1.4
carrier [ppm]	118.2	118.2	99
transfer 2	NCA-DCP	NCA-DCP	NCO-DCP
field [kHz]	38(N) 22.8(C) 100(H)	38(N) 22.8(C) 83.3(H)	38(N) 53.2(C) 83.3(H)
shape (ramp)	90.100 (C)	90.100 (C)	90.100 (C)
contact/mixing time [ms]	4.6	4.2	4.6
carrier [ppm]	57.6	57.6	165
transfer 3	DARR	DARR	DARR
field [kHz]	14.1 (H)	11.9 (H)	11.8 (H)
contact/ mixing time [ms]	50	50	400
carrier [ppm]	57.6	57.6	165
t1 increments	128	120	104
spectral width [kHz]	3.04	3.04	7.6
aqu. time [ms]	21	19.7	6.8
t2 increments	3390	3390	3390
spectral width [kHz]	100	100	100
aqu. time [ms]	17	17	17
^{1}H SPINAL decoupling [kHz]	100	83.3	83.3
number of scans	1464	1600	2960
total measurement time	6d17h	2d21h	7d6h

dimensionality	3D							
**experiment**	**NCACX**				**NCOCX**		**CONCA**	
**figure**	Fig. 2a, 5	not shown	Fig. 5c	not shown	Fig. 2a	not shown	Fig. 2a	not shown
**sample**	U-^{13}C,^{15}N-DGK	U-^{13}C,^{15}N-DGK-**I,LV**	U-^{13}C,^{15}N-DGK + **AMP-PCP**	U-^{13}C,^{15}N-DGK + **DOG**	U-^{13}C,^{15}N-DGK	U-^{13}C,^{15}N-DGK-**I,LV**	U-^{13}C,^{15}N-DGK	U-^{13}C,^{15}N-DGK-**I,LV**
**probehead**	HCN	E-free	E-free	HCN	HCN	E-free	HCN	E-free
**recycle delay [s]**	2.5	0.8	1.0	2.5	2.5	1.0	3.0	1.0
**transfer 1**	HN-CP	HN-CP	HN-CP	HN-CP	HN-CP	HN-CP	HC-CP	HC-CP
**field [kHz]**	74.1(H) 41.7(N)	60.4(H) 41.7(N)	74.3(H) 41.7(N)	74.7(H) 41.7(N)	72.8(H) 41.7(N)	60.4(H) 41.7(N)	74.1(H) 55.6©	77.1(H) 55.6©
**shape (ramp)**	80.100 (H)	80.100 (H)	80.100 (H)	80.100 (H)	80.100 (H)	80.100 (H)	80.100 (H)	80.100 (H)
**contact time [ms]**	1.80	0.95	1.40	1.40	1.25	0.95	1.50	1.70
**carrier [ppm]**	118.2	118.2	118.2	118.2	118.2	118.2	176.6	176.6
**transfer 2**	NCA-DCP	NCA-DCP	NCA-DCP	NCA-DCP	NCO-DCP	NCO-DCP	CON-DCP	CON-DCP
**field [kHz]**	38(N) 22.8© 100(H)	38(N) 22.8© 83.3(H)	38(N) 22.8© 83.3(H)	38(N) 22.8© 100(H)	38(N) 53.2© 100(H)	38(N) 53.2© 83.3(H)	38(N) 53.2© 100(H)	38(N) 53.2© 83.3(H)
**shape (ramp)**	90.100 (C)	90.100 (C)	90.100 (C)	90.100 (C)	90.100 (C)	90.100 (C)	90.100 (C)	90.100 (C)
**contact/mixing time [ms]**	4.0	4.7	4.6	4.5	4.0	4.8	8.0	7.1
**carrier [ppm]**	57.6	57.6	57.6	57.6	176.6	176.6	118.2	118.2
**transfer 3**	DARR	DARR	DARR	DARR	DARR	DARR	NCA-DCP	NCA-DCP
**field [kHz]**	12.5 (H)	11.8 (H)	11.9 (H)	13.6 (H)	12.5 (H)	11.8 (H)	38(N) 22.8© 100(H)	38(N) 22.8© 83.3(H)
**shape (ramp)**							90.100 (C)	90.100 (C)
**contact/ mixing time [ms]**	50	50	50	50	100	100	5.0	4.8
**carrier [ppm]**	57.6	57.6	57.6	57.6	176.6	176.6	57.6	57.6
**t1 increments**	80	74	80	80	64	64	40	46
**spectral width [kHz]**	3.04	3.04	3.04	3.04	3.04	3.04	2.5	2.5
**aqu. time [ms]**	13.2	12.2	13.2	13.2	10.5	10.5	7.9	9.1
**t2 increments**	64	94	72	64	50	50	48	50
**spectral width [kHz]**	5.1	6.6	5,07	5,07	2.5	2.5	2.5	2.5
**aqu. time [ms]**	6.3	7.1	7.1	6.3	9.9	9.9	9.5	9.9

**t3 increments**	3390	3390	3390	3390	3390	3390	2988	2988
**spectral width [kHz]**	100	100	100	100	100	100	100	100
**aqu. time [ms]**	17	17	17	17	17	17	15	15
**^{1}H SPINAL decoupling [kHz]**	100	83.3	83.3	100	100	83.3	100	83.3
**number of scans**	64	136	88	88	72	152	96	240
**total measurement time**	9d19h	9d16h	6d9h	13d12h	7d1h	6d3h	6d12h	6d16h

References

1 Badola, P. & Sanders, C. R. Escherichia coli diacylglycerol kinase is an evolutionarily optimized membrane enzyme and catalyzes direct phosphoryl transfer. *J. Biol. Chem.* **272**, 24176-24182 (1997).

2 Ullrich, S. J., Hellmich, U. A., Ullrich, S. & Glaubitz, C. Interfacial enzyme kinetics of a membrane bound kinase analyzed by real-time MAS-NMR. *Nat. Chem. Biol.* **7**, 263-270, doi:10.1038/nchembio.543 (2011).

3 Szeverenyi, N. M., Sullivan, M. J. & Maciel, G. E. Observation of spin exchange by two-dimensional fourier-transform C-13 cross polarization-magic-angle spinning. *J. Magn. Reson.* **47**, 462-475 (1982).

4 Chen, Y. *et al.* Conformation and topology of diacylglycerol kinase in E.coli membranes revealed by solid-state NMR spectroscopy. *Angew. Chem. Int. Ed. Engl.* **53**, 5624-5628, doi:10.1002/anie.201311203 (2014).

5 Vranken, W. F. *et al.* The CCPN data model for NMR spectroscopy: development of a software pipeline. *Proteins* **59**, 687-696, doi:10.1002/prot.20449 (2005).

6 Luca, S. *et al.* Secondary chemical shifts in immobilized peptides and proteins: a qualitative basis for structure refinement under magic angle spinning. *J. Biomol. NMR* **20**, 325-331 (2001).

7 Li, D. *et al.* Crystal structure of the integral membrane diacylglycerol kinase. *Nature* **497**, 521-524, doi:10.1038/nature12179 (2013).

8 Li, D. *et al.* Ternary structure reveals mechanism of a membrane diacylglycerol kinase. *Nature communications* **6**, 10140, doi:10.1038/ncomms10140 (2015).

9 Lau, F. W. & Bowie, J. U. A method for assessing the stability of a membrane protein. *Biochemistry* **36**, 5884-5892, doi:10.1021/bi963095j (1997).
